# Cross-sectional and longitudinal associations between empirically derived dietary patterns and frailty among older men: The Concord Health and Ageing in Men Project

**DOI:** 10.1016/j.jnha.2023.100021

**Published:** 2024-01-01

**Authors:** Rebecca Luong, Rosilene Ribeiro, Vasi Naganathan, Fiona Blyth, Louise M Waite, David J Handelsman, David G Le Couteur, Markus J Seibel, Vasant Hirani

**Affiliations:** a Nutrition and Dietetics Group, Sydney Nursing School, Faculty of Medicine and Health, The University of Sydney, NSW, Australia; b ARC Centre of Excellence in Population Ageing Research (CEPAR), The University of Sydney, NSW, Australia; c Charles Perkins Centre, The University of Sydney, NSW, Australia; d School of Life and Environmental Sciences, Faculty of Science, The University of Sydney, NSW, Australia; e Centre for Education and Research on Ageing, Concord Hospital, The University of Sydney, Concord, NSW, Australia; f Concord Clinical School, Faculty of Medicine and Health, The University of Sydney, Concord, NSW, Australia; g School of Public Health, The University of Sydney, NSW, Australia; h ANZAC Research Institute, The University of Sydney, Concord, NSW, Australia; i Andrology Department, Concord Hospital, Concord, NSW, Australia

**Keywords:** Diet, Frailty syndrome, Older men, Food

## Abstract

**Background:**

Diet may be associated with frailty.

**Objective:**

We aimed to evaluate the associations between empirically derived dietary patterns and frailty in older men.

**Design:**

Prospective cohort study.

**Setting:**

The Concord Health and Ageing in Men Project, Sydney, Australia.

**Participants:**

785 community-dwelling older Australian men aged 75 years and older.

**Methods:**

Men underwent dietary assessment using a validated dietitian-administered diet history questionnaire. Factor analysis identified three dietary patterns. Multinomial logistic regression was conducted between frailty and dietary patterns for cross-sectional analyses and longitudinal analyses over a 3-year follow-up. Frailty was defined by the Fried frailty phenotype.

**Results:**

Of the 785 men, pre-frailty was prevalent in 47.1% (n = 370), and frailty in 8.3% (n = 65). In fully adjusted cross-sectional analyses, the top tertile and a higher ‘vegetables-legumes-seafood’ dietary pattern score were associated with reduced prevalence of frailty (OR 0.34 [95% CI: 0.12, 0.93, P = .036]) and OR 0.50 [95% CI: 0.30, 0.83, P = .007] respectively). The top tertile of the ‘discretionary-starchy vegetables-processed meats’ dietary pattern was also associated cross-sectionally with increased prevalence of pre-frailty (OR 1.75 [95% CI: 1.08, 2.83, P = .022]). Of the 296 robust men in fully adjusted longitudinal analyses, the incidence of pre-frailty was 52.4% (n = 155), and frailty was 5.4% (n = 16) over a 3-year follow-up. The middle tertile of the ‘vegetables-legumes-seafood’ dietary pattern had a non-significant trend towards reduced incident pre-frailty (OR 0.52 [95% CI: 0.27, 1.00, P = .050]).

**Conclusion:**

Consumption of a ‘vegetables-legumes-seafood’ dietary pattern appears to be less favoured by frail older men.

## 1 Introduction

Frailty is an extreme consequence of the normal ageing process characterised by a decline in multiple physiological functions, associated with increased vulnerability to adverse health outcomes and increased healthcare costs [1,2]. There is a higher prevalence of frailty in old age and it is a major risk factor for morbidity, disability, and mortality [1,2]. Older adults are also susceptible to the anorexia of ageing including loss of appetite, reduced food intake, and nutritional inadequacies that are potential modifiable risk factors of frailty [1]. Dietary patterns are composites of foods, nutrients, and other dietary factors, and is representative of the synergistic effects on health rather than the single effects from individual dietary components [3].

Dietary patterns are derived from three main approaches [4]: *a posteriori*, *a priori* and hybrid. The *a posteriori* or data-driven approach involves empirically deriving dietary patterns from the diets consumed in the cohort by identifying factors that explain variation in the dietary pattern such as through cluster, principal component, or exploratory factor analysis [4,5]; the *a priori* or hypothesis-driven approach uses pre-established diet quality scores, or indices created based on current evidence of ideal dietary patterns and their health relation [4,5]; and the hybrid approach combines the *a posteriori* and *a priori* approaches [4,5]. Most studies have used *a priori* approaches to examine associations between dietary patterns and frailty, with only a limited number of studies investigating the relationship between *a posteriori* empirically derived dietary patterns and frailty in older adults [6]. The main advantage of *a posteriori* approach is that it is independent of prior knowledge, takes into account multiple dimensions, provides valuable insights into the interrelationships between food combinations and the habitual dietary patterns adopted by individuals [5].

The Concord Health and Ageing in Men Project (CHAMP) is a prospective cohort study investigating the causes and consequences of major geriatric syndromes in men. Men were the focus of the CHAMP study because epidemiological studies of ageing tended to include less men, and men have a shorter life expectancy [7]. Through the CHAMP cohort, it has been previously shown that the *a priori* revised Dietary Guideline Index was cross-sectionally associated with reduced prevalence of frailty but was not associated with the prevalence of pre-frailty in older men [8]. The aim was to evaluate cross-sectional and longitudinal associations between empirically derived dietary patterns and frailty in older men aged 75 years and over.

## 2 Methods

### 2.1 Study participants

**Fig. 1:**
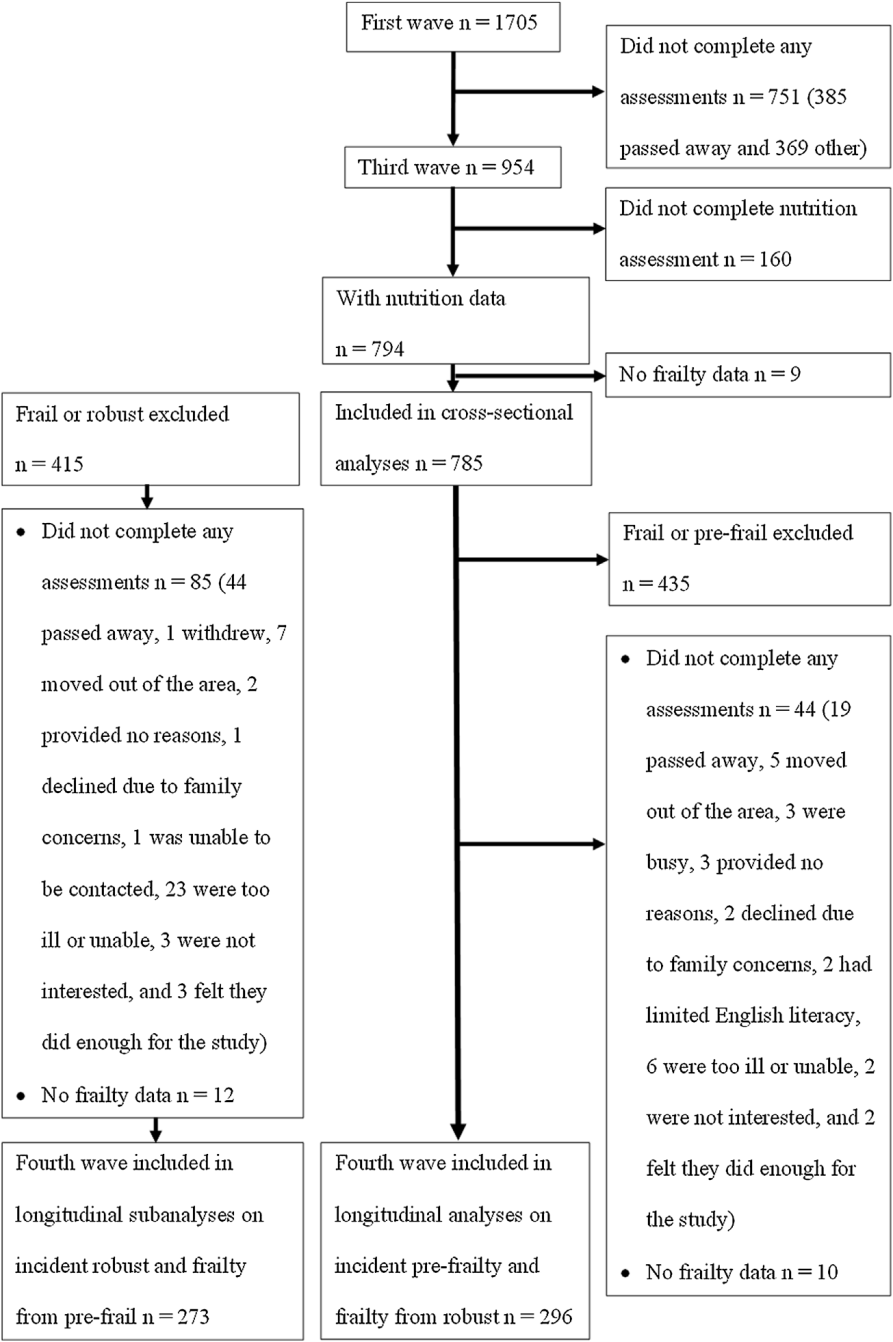
Flow diagram of participants included in cross-sectional and longitudinal analyses.

Participants were recruited from a defined geographic region of three Local Government Areas around Concord Hospital in Sydney (Burwood, Canada Bay and Strathfield) using the New South Wales electoral roll in which enrolment is compulsory in Australia. The only exclusion criteria was living in a residential aged care facility. Invitation letters were sent. For men who did not respond one week later, those with a listed telephone number were called, and those without a listed telephone number were sent a second invitation letter. Of the 2815 eligible men, 1511 (54%) agreed to participate. Additionally, 194 eligible men volunteered independently of the invitation letters as they were informed by friends or reports on the study in the local newspapers. A total of 1705 men aged 70 years and over were recruited in the first wave (between January 2005 and June 2007) [7]. Dietary data were first collected in the third wave of CHAMP (between August 2010 and August 2013, baseline in the present study) involving 794 men aged 75 years and over. Of these, 785 men (98.9%) also had frailty data and were included in the cross-sectional analyses. The fourth wave of CHAMP (between August 2014 and June 2016, 3-year follow-up in this study) also included frailty data collection in the men then aged 78 years and over. Men who were frail and pre-frail at baseline were excluded in longitudinal analyses from robust, and men who were robust and frail at baseline were excluded in longitudinal sub-analyses from pre-frail. Of the 350 men who were robust at baseline, 296 men (84.6%) had frailty data at 3-year follow-up and were included in the longitudinal analyses. Of the 370 men who were pre-frail at baseline, 273 men (73.8%) had frailty data at 3-year follow-up and were included in longitudinal subanalyses. Flowchart of participants’ inclusion in cross-sectional, longitudinal, and longitudinal subanalyses is shown in Fig. 1. The CHAMP study was conducted according to the guidelines laid down in the Declaration of Helsinki and all procedures involving human subjects were approved by the Concord Hospital Human Research Ethics Committee (HREC/14/CRGH/17). Written informed consent was obtained from all participants.

### 2.2 Dietary intake

Dietary data was collected using a validated dietitian-administered diet history questionnaire at baseline. Participants were asked about their usual dietary intake in the previous 3 months and food models, photographs, and household measures were used to assist with estimation of amounts consumed [8,9]. A food checklist was included, and relatives, carers, and/or family members of participants were encouraged to attend the interview to aid memory recall [9]. The validity of this diet history questionnaire by comparison with a prospective 4-day weighed food record was previously reported in a subgroup of 56 CHAMP men [9]. The mean differences in dietary intakes between the two methods were generally less than 20% [9].

Dietary data was initially analysed using FoodWorks 7 Professional for Windows (Xyris Software (Brisbane, Australia) Pty Ltd) that utilised The Australian Food, Supplement and Nutrient Database 2007 (AUSNUT 2007) [10]. A matching file was used to update the dietary data to The Australian Food, Supplement and Nutrient Database 2011–2013 (AUSNUT 2011–2013) and entries were converted to nutrients, foods, and food groups through FoodWorks [10]. A serve of each food group was based on the Australian Guide to Healthy Eating [11,12]. Discretionary foods are not necessary to provide the nutrients the body needs, are generally high in saturated fats, sugars, salt and/or alcohol, and thus are energy-dense and nutrient-poor according to the Australian Dietary Guidelines [13]. Due to the different classification of sugar, solid fat, and alcohol as food groups in FoodWorks, a serve of discretionary was defined as 4.2 g (1 teaspoon) sugar, 4.8 g (1 teaspoon) solid fat equivalents, or 10 g alcohol (1 standard drink) [8,12].

Dietary pattern exposure was defined as dietary intakes at baseline nutrition. The 23 food groups include: (i) refined grains; (ii) wholegrains; (iii) citrus, melons and berries; (iv) other fruit; (v) fruit juice; (vi) dark green vegetables; (vii) red orange vegetables (viii) starchy vegetables; (ix) other vegetables; (x) legumes; (xi) red meats; (xii) poultry; (xiii) eggs; (xiv) processed meats; (xv) organ meats; (xvi) seafood; (xvii) nuts and seeds; (xviii) soy products; (xix) milk (xx) cheese; (xxi) yoghurt; (xxii) milk alternatives; (xxiii) discretionary. Other vegetables are vegetables that are not dark green, red orange, or starchy. Other fruits are fruits other than citrus, melons and berries, or fruit juice.

### 2.3 Frailty measurement

Frailty data was collected at baseline and at 3-year follow-up. Frailty was defined using the Fried frailty phenotype criteria according to the Cardiovascular Health Study (CHS) for weakness and slowness [14], and adapted criteria for weight loss, exhaustion, and low activity due to unavailability of the exact measurement [15]. Weakness was defined as being in the lowest sample quintile for grip strength adjusted for body mass index (BMI), measured by the mean value of two trials on each side using a Jamar dynamometer. Slowness was defined as being in the lowest sample quintile for walking speed adjusted for height, measured as the mean value of two trials on a six-metre course at usual pace. Weight loss was indicated if a participant’s current weight (measured at the clinic visit) was lower by 15% or more than self-reported heaviest weight reported at first wave (or lower than weight at 25 years old reported at first wave, if missing data on heaviest weight). Exhaustion was defined by participants reporting either ‘a little of the time’ or ‘none of the time’ on the Short-form Health Survey (SF-12) on ‘how much of the time during that past 4 weeks did you have a lot of energy?’ [16]. Low physical activity was indicated as being in the lowest sample quintile of activity as measured by the Physical Activity Scale for the Elderly (PASE) (a score of 72 or lower among CHAMP participants) [17]. Participants were classified as robust if they had none, pre-frail if they had one or two, and frail if they had three or more of the following components: weakness, slowness, weight loss, exhaustion, and low activity.

### 2.4 Other measurements

Data on anthropometry, socio-demographics, lifestyle, and health factors were collected through self-reported or interviewer-administered questionnaires, biochemical analyses, medication inventory, and physical measurements at baseline. Height, weight, waist circumference, and hip circumference were measured following standardised protocols as previously described [8]. BMI was calculated as kg/m^2^. Physical activity was assessed through PASE questionnaire.

Marital status was categorised into ‘married/de facto’ and ‘not married/divorced/separated/widowed/never married/other’. ‘Age Pension only’ referred to those who only received the Age Pension, whilst ‘other’ referred to those with other sources of income apart from the Age Pension including veteran pension, repatriation pension, superannuation, private income, business ownership, farm ownership, business partnership, wage, salary, and/or other. Country of birth was categorised into ‘Australia’, ‘Greece/Italy’, and ‘other’. Smoking status was categorised into ‘nonsmoker’, ‘ex-smoker’, and ‘current smoker’ based on self-reported smoking history.

Prescription and non-prescription medication used daily or almost daily were brought to the baseline clinic visit and recorded. Participants were asked whether they had taken any other medications during the past month. Reported medications were coded using the Iowa Drug Information Service drug code numbers. Supplement use including vitamins, minerals, and/or fish oil was categorised into ‘yes’ and ‘no’.

Anaemia was defined as haemoglobin levels <130 g/L [18]. The inflammatory biomarker included was cytokine interleukin-6 (IL-6). Self-rated health was categorised into ‘very poor/poor/fair’ and ‘good/excellent’ [16]. The number of comorbidities was determined by the sum of all conditions that participants reported including: diabetes, thyroid disease, osteoporosis, Paget’s disease, stroke, Parkinson’s disease, kidney stones, dementia, depression, epilepsy, hypertension, myocardial infarction, angina, congestive cardiac failure, intermittent claudication, chronic obstructive pulmonary disease, liver disease, renal disease, arthritis, gout, and cancer (excluding nonmelanoma skin cancers).

### 2.5 Dietary pattern analysis

Factor analysis using orthogonal transformation, varimax rotation with the 23 food groups as variables was conducted for the extraction of the dietary pattern factors. Factors were retained based on an eigenvalue >1.5, a breakpoint in the scree plot, and the interpretability [19]. Satisfactory factor analysis was determined through the Kaiser–Meyer–Olkin measure (>0.5 is acceptable) and the Bartlett’s test (P < 0.05 as statistically significant) [19]. Loadings of each food group represented the correlation between the food groups and the factor. Major contributions were determined by an absolute value of factor loading >0.30 [20,21]. Positive loadings represented alignment to the dietary pattern and negative loadings indicated non-alignment to that pattern. The higher the factor score for each dietary pattern indicated greater conformity with the dietary pattern for that individual.

### 2.6 Statistical analysis

Statistical analysis was carried out using SPSS software version 25 (IBM Corp., Armonk, NY, USA) [22]. Normality tests (histogram, Q-Q plot and Shapiro-Wilk test) conducted found that most data had a skewed distribution. Thus, descriptive characteristics were expressed as median (interquartile range) (IQR) and as number of participants (percentage of participants). Participant characteristics at nutrition assessment according to dietary pattern scores were compared through non-parametric tests including chi-square tests, and median tests with Bonferroni correction for multiple testing.

The associations between dietary pattern scores with pre-frailty and frailty in cross-sectional analyses, with incident pre-frailty and frailty from robust in longitudinal analyses, and with incident robust and frailty from pre-frail in longitudinal subanalyses, were evaluated through multinomial logistic regression. The outcomes of interest for cross-sectional analyses were: robust versus pre-frail versus frail. Longitudinal analyses from robust excluded participants who were pre-frail or frail at baseline, and had the following outcomes of interest: participants remaining robust versus those who deteriorated to pre-frail versus those who deteriorated to frail at 3-year follow-up. Longitudinal subanalyses from pre-frail excluded participants who were robust or frail at baseline, and had the following outcomes of interest: participants remaining pre-frail versus those who improved to robust versus those who deteriorated to frail at 3-year follow-up. Dietary pattern scores were analysed as both continuous and categorical variables (i.e. categorised into three tertiles with bottom as the reference category). Continuous and categorical results are presented as odds ratios (ORs) with 95% confidence intervals.

Pre-specified potential confounders considered for inclusion in the multinomial logistic regression models were socio-demographic and lifestyle factors (age, BMI, country of birth, marital status, age pension, smoking status, energy intake, and supplement use including vitamins, minerals, and/or fish oil, and health (haemoglobin, IL-6, number of medications, self-rated health, and number of comorbidities). Physical activity was not considered for inclusion in the model since it is one of the components of frailty. Backward stepwise selection with likelihood ratio tests was used to build models containing dietary pattern factor scores as the explanatory variable against the outcomes, and was confirmed using forward stepwise selection. Covariates included in the multivariable model were: age (continuous), BMI (continuous), smoking status (nonsmoker v. ex-smoker v. current smoker), energy intake (continuous), supplement use including vitamins, minerals, and/or fish oil (yes v. no), haemoglobin (continuous), number of medications (continuous), number of comorbidities (continuous), and self-rated health (very poor/poor/fair v. good/excellent). A statistically significant Likelihood Ratio chi-square test and statistically non-significant Pearson and Deviance tests indicated goodness of fit of the finally adjusted models. Collinearity diagnostics was conducted and there was no collinearity in the models with variance inflation factors (VIFs) <2.5 [23]. Participants with missing data on covariates were excluded from the adjusted models.

## 3 Results

### 3.1 Dietary patterns

**Fig. 2:**
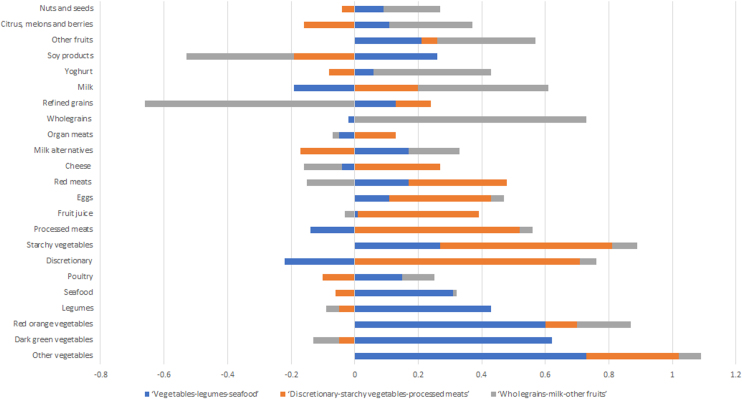
Factor loadings with varimax rotation to determine the association between 23 food groups and factors representing dietary patterns among older Australian men (n = 785).

Factor analysis identified three main dietary patterns for the population in the present study (scree plot shown in Supplementary Fig. 1). Food groups and factor loadings for each dietary pattern are presented in Fig. 2 (absolute factor loadings, % variance explained, and eigenvalues are shown in Supplementary Table 1). Dietary patterns were named according to major contributing food groups with high factor loadings: ‘vegetables-legumes-seafood’, ‘discretionary-starchy vegetables-processed meats’, and ‘wholegrains-milk-other fruits’. These dietary patterns respectively accounted for 8.71%, 7.66%, and 7.32% of the variance in food intakes, explaining a total of 23.7% of the variability.

### 3.2 Participant characteristics

Table 1 presents the participant characteristics according to tertiles of dietary pattern scores. A total of 785 men had dietary intake and frailty data available at nutrition assessment. Participants’ median age was 80.0 (IQR 77.0−84.0) years and BMI was 27.5 (IQR 25.0−30.2) kg/m^2^ (Table 1). Characteristics that differed between the tertiles included BMI, waist circumference, hip circumference, marital status, source of income, country of birth, and IL-6. Supplementary Table 2 presents dietary intake according to tertiles of dietary pattern scores. As expected food group intakes and thus energy and nutrient intakes were also different between the dietary pattern tertiles. Fruit juice, milk alternatives, organ meats, yoghurt, and soy products were minimally consumed by all participants.

**Table 1:** Participant characteristics (median and interquartile range; percentages and number of participants) according to dietary pattern factor score tertiles (n = 785).

		Factor 1: ‘Vegetables-legumes-seafood’				Factor 2: ‘Discretionary-starchy vegetables-processed meats’				Factor 3: ‘Wholegrains-milk-other fruits’			
Variables	All	Bottom	Middle	Top	P value^1^	Bottom	Middle	Top	P value^1^	Bottom	Middle	Top	P value^1^
	n = 785	n = 262	n = 262	n = 261		n = 262	n = 262	n = 261		n = 262	n = 262	n = 261	
Age (years)	80.0 (77.0−84.0)	81.0 (78.0−85.0)^a,b^	81.0 (77.0−83.0)^a,c^	79.0 (77.0−83.0)^b.c^	.76^a^	80.0 (77.0−84.0)	80.5 (77.0−84.0)	80.0 (78.0−84.0)	.66	80.0 (77.0−83.0)	81.0 (78.0−84.0)	80.0 (77.0−84.0)	.15
					.022^b^								
					.047^c^								
BMI (kg/m^2^) (n = 782)	27.5 (25.0−30.2)	27.4 (25.2−30.1)^a,b^	28.3 (25.5−30.8)^a,c^	26.7 (24.5−29.7)^b,c^	.13^a^	27.4 (25.4−30.3)	27.4 (24.9−30.1)	27.6 (24.9−30.1)	.83	27.5 (25.2−30.1)	27.9 (25.5−30.6)	26.9 (24.6−29.6)	.22
					.26^b^								
					.010^c^								
Waist circumference (cm)	101.5 (94.1−108.3)	102.6 (95.8−108.7)^a,b^	102.7 (95.8−109.9)^a,c^	98.9 (92.5−106.5)^b,c^	1.00^a^	100.6 (93.7−107.7)	102.4 (94.0−108.6)	101.5 (95.0−108.8)	.38	100.5 (93.2−107.6)	102.8 (95.8−109.5)	101.2 (94.0−107.9)	.10
					.004^b^								
					.013^c^								
Hip circumference (cm)	102.0 (97.5−108.0)	102.9 (97.8−108.2)^a,b^	102.8 (98.3−109.3)^a,c^	100.7 (96.0−106.0)^b,c^	1.00^a^	102.0 (97.0−107.1)	102.4 (98.0−108.3)	101.9 (97.6−108.2)	.79	101.0 (96.8−107.5)	102.3 (98.5−108.8)	102.0 (97.1−107.9)	.50
					.038^b^								
					.048^c^								
Marital status (n = 779)					.008				.77				.21
Married/de facto	592 (76.0)	183 (70.1)	198 (76.2)	211 (81.8)		200 (77.2)	199 (76.2)	193 (74.5)		206 (79.5)	197 (75.5)	189 (73.0)	
Not married/divorced/separated/widowed/never married/other	187 (24.0)	78 (29.9)	62 (23.8)	47 (18.2)		59 (22.8)	62 (23.8)	66 (25.5)		53 (20.5)	64 (24.5)	70 (27.0)	
Source of income (n = 783)					.15				.73				<.001
Age Pension Only	314 (40.1)	108 (41.4)	114 (43.5)	92 (35.4)		108 (41.4)	100 (38.2)	106 (40.8)		138 (52.9)	101 (38.5)	75 (28.8)	
Other	469 (59.9)	153 (58.6)	148 (56.5)	168 (64.6)		153 (58.6)	162 (61.8)	154 (59.2)		123 (47.1)	161 (61.5)	185 (71.2)	
Country of birth					<.001				<.001				<.001
Australia	412 (52.5)	155 (59.2)	145 (55.3)	112 (42.9)		103 (39.3)	153 (58.4)	156 (59.8)		92 (35.1)	137 (52.3)	183 (70.1)	
Greece/Italy	186 (23.7)	41 (15.3)	69 (26.3)	76 (29.1)		88 (33.6)	50 (19.1)	48 (18.4)		101 (38.5)	63 (24.0)	22 (8.4)	
Other	187 (23.8)	66 (25.2)	48 (18.3)	73 (28.0)		71 (27.1)	59 (22.5)	57 (21.8)		69 (26.3)	62 (23.7)	56 (21.5)	
Cigarette smoking status (n = 778)					0.18				0.12				0.13
Nonsmoker	316 (40.6)	108 (41.5)	93 (35.6)	115 (44.7)		105 (40.5)	114 (43.7)	97 (37.6)		96 (36.8)	108 (41.9)	112 (43.2)	
Ex-smoker	434 (55.8)	141 (54.2)	161 (61.7)	132 (51.4)		149 (57.5)	139 (53.3)	146 (56.6)		150 (57.5)	142 (55.0)	142 (54.8)	
Current smoker	28 (3.6)	11 (4.2)	7 (2.7)	10 (3.9)		5 (1.9)	8 (3.1)	15 (5.8)		15 (5.7)	8 (3.1)	5 (1.9)	
Supplement use (vitamins, minerals and/or fish oil)	165 (21.0)	55 (21.0)	59 (22.5)	51 (19.5)	.71	58 (22.1)	58 (22.1)	49 (18.8)	.55	58 (22.1)	50 (19.1)	57 (21.8)	.64
Number of medications	4.0 (3.0−6.0)	5.0 (3.0−7.0)^a,b^	5.0 (3.0−7.0)^a,c^	4.0 (2.0−6.0)^b,c^	.64^a^	4.0 (3.0−6.0)	5.0 (3.0−7.0)	4.0 (3.0−6.0)	.29	4.0 (2.0−7.0)	4.0 (3.0−6.0)	4.0 (3.0−6.5)	.71
					.075^b,c^								
Anaemia (n = 759)	132 (17.4)	52 (20.5)	42 (16.8)	38 (14.9)	.24	46 (18.3)	39 (15.3)	47 (18.6)	.55	46 (18.3)	46 (17.9)	40 (16.0)	.77
Haemoglobin (g/L) (n = 759)	143.0 (134.0−152.0)	141.0 (132.0−151.3)	143.5 (135.0−151.0)	144.0 (135.0−152.0)	.37	142.0 (132.0−151.0)	144.0 (136.0−152.0)	144.0 (134.0−152.0)	.20	142.0 (132.0−151.8)	143.0 (135.0−151.0)	143.0 (135.0−152.0)	.96
Interleukin-6 (pg/mL) (n = 716)	2.5 (1.3−4.8)	2.9 (1.5−5.6)^a,b^	2.5 (1.2−4.4)^a,c^	2.2 (1.2−4.1)^b,c^	.39^a^	2.1 (1.1−4.6)^a,b^	2.7 (1.4−5.0)^a,c^	2.9 (1.4−5.0)^b,c^	.092^a^	2.8 (1.4−5.0)	2.3 (1.3−4.0)	2.7 (1.3−5.0)	.29
					.011^b^				.015^b^				
					.52^c^				1.00^c^				
Self-rated health (n = 784)					.060				.55				.20
Very poor/poor/fair	201 (25.6)	74 (28.2)	74 (28.2)	53 (20.4)		69 (26.4)	61 (23.3)	71 (27.2)		77 (29.5)	64 (24.4)	60 (23.0)	
Good/excellent	583 (74.4)	188 (71.8)	188 (71.8)	207 (79.6)		192 (73.6)	201 (76.7)	190 (72.8)		184 (70.5)	198 (75.6)	201 (77.0)	
Number of comorbidities (n = 784)	2.0 (1.0−3.0)	2.0 (1.0−4.0)	2.0 (2.0−3.0)	2.0 (1.0−3.0)	.41	2.0 (1.0−4.0)	2.0 (1.0−4.0)	2.0 (1.0−3.0)	.22	2.0 (1.0−3.0)	2.0 (1.0−4.0)	2.0 (1.0−3.0)	.24
PASE (n = 784)	121.0 (77.1−161.7)	114.6 (64.9−162.1)	120.8 (73.1−155.7)	131.0 (89.3−162.2)	.13	123.7 (76.4−158.7)	117.8 (64.9−157.4)	120.6 (85.9−165.9)	.71	117.8 (76.8−155.1)	131.2 (74.0−166.7)	118.6 (80.3−157.4)	.37

*Notes*: BMI = body mass index; PASE = Physical Activity Scale for the Elderly.

1 P values were obtained using the median test and Bonferroni correction for multiple tests to compare all dietary pattern score tertile groups for differences in median values of continuous variables. Differences between groups are denoted by each letter a, b, or c. P values were obtained using the chi-square test to compare all dietary pattern score tertile groups for differences in proportions of participants in categories for categorical variables.

For the ‘vegetables-legumes-seafood’ dietary pattern, the top tertile had higher intakes of other vegetables, dark green vegetables, red orange vegetables, seafood, poultry, and nuts and seeds, and lower intakes of discretionary, and processed meats than both bottom and middle tertiles. The top tertile of ‘vegetables-legumes-seafood’ dietary pattern also had higher intakes of legumes, starchy vegetables, other fruits, and eggs, and lower intakes of milk than the bottom tertile.

For the ‘discretionary-starchy vegetables-processed meats’ dietary pattern, the top tertile had higher intakes of discretionary, starchy vegetables, processed meats, eggs, red meats, other vegetables, cheese, and milk than both bottom and middle tertiles. The top tertile of ‘discretionary-starchy vegetables-processed meats’ dietary pattern also had higher intakes of refined grains than the bottom tertile.

For the ‘wholegrains-milk-other fruits’ dietary pattern, the top tertile had higher intakes of wholegrains, milk, nuts and seeds, and discretionary, and lower intakes of refined grains than both bottom and middle tertiles. The top tertile of the ‘wholegrains-milk-other fruits’ dietary pattern also had higher intakes of other fruits, citrus, melons and berries, red orange vegetables, and starchy vegetables, and lower intakes of red meats than the bottom tertile.

### 3.3 Cross-sectional analyses of associations between dietary patterns, prevalence of pre-frailty and frailty

Of the 785 men, the prevalence of pre-frailty was 47.1% (n = 370) and frailty was 8.3% (n = 65). Cross-sectional analyses evaluating associations between dietary pattern scores and frailty status are shown in Table 2. Compared to the bottom tertile, the top tertile and a higher ‘vegetables-legumes-seafood’ dietary pattern score were associated with reduced prevalence of frailty in unadjusted analyses (OR 0.21 [95% CI: 0.10, 0.45, P < .001]) and OR 0.45 [95% CI: 0.31, 0.67, P < .001]), which remained in fully adjusted analyses (OR 0.34 [95% CI: 0.12, 0.93, P = .036]) and OR 0.50 [95% CI: 0.30, 0.83, P = .007]). A higher ‘vegetables-legumes-seafood’ dietary pattern score was associated with reduced prevalence of pre-frailty in unadjusted analyses (OR 0.84 [95% CI: 0.72, 0.97, P = .021]), but not in the fully adjusted analyses. The ‘vegetables-legumes-seafood’ dietary pattern score as categorical variables was not associated with the prevalence of pre-frailty in unadjusted and fully adjusted analyses.

**Table 2:** Cross-sectional associations between dietary pattern factor scores, the prevalence of pre-frailty and frailty using multinomial logistic regression presented as odds ratios (n = 785).

Dietary Pattern Factor Scores	Bottom tertile (reference category)	Middle tertile	Top tertile	As continuous variable (per 1 increment)
Factor 1: ‘Vegetables-legumes-seafood’^a^				
**Pre-frailty**				
Model 1	1	0.94 (0.66, 1.36) P = .75	0.75 (0.52, 1.08) P = .12	0.84 (0.72, 0.97) P = .021
Model 2	1	1.03 (0.70, 1.51) P = .87	0.92 (0.62, 1.34) P = .65	0.90 (0.77, 1.05) P = .18
Model 3	1	1.11 (0.74, 1.66) P = .62	1.03 (0.69, 1.54) P = .90	0.91 (0.78, 1.07) P = .28
**Frailty**				
Model 1	1	0.60 (0.33, 1.08) P = .090	0.21 (0.10, 0.45) P < .001	0.45 (0.31, 0.67) P < .001
Model 2	1	0.90 (0.46, 1.78) P = .77	0.27 (0.11, 0.66) P = .004	0.55 (0.36, 0.85) P = .006
Model 3	1	0.99 (0.44, 2.21) P = .98	0.34 (0.12, 0.93) P = .036	0.50 (0.30, 0.83) P = .007
Factor 2: ‘Discretionary-starchy vegetables-processed meats’^b^				
**Pre-frailty**				
Model 1	1	1.13 (0.79, 1.61) P = .52	1.28 (0.90, 1.83) P = .17	1.02 (0.88, 1.18) P = .84
Model 2	1	1.22 (0.83, 1.80) P = .32	1.58 (1.00, 2.49) P = .048	1.11 (0.92, 1.34) P = .28
Model 3	1	1.29 (0.85, 1.94) P = .23	1.75 (1.08, 2.83) P = .022	1.07 (0.87, 1.31) P = .52
**Frailty**				
Model 1	1	1.06 (0.57, 1.97) P = .85	0.79 (0.40, 1.54) P = .48	0.96 (0.73, 1.26) P = .77
Model 2	1	1.40 (0.66, 2.94) P = .38	1.49 (0.59, 3.72) P = .40	1.47 (1.02, 2.11) P = .038
Model 3	1	1.34 (0.57, 3.15) P = .51	1.72 (0.61, 4.84) P = .30	1.20 (0.81, 1.77) P = .37
Factor 3: ‘Wholegrains-milk-other fruits’^c^				
**Pre-frailty**				
Model 1	1	1.03 (0.72, 1.48) P = .86	0.94 (0.65, 1.34) P = .72	1.01 (0.88, 1.17) P = .85
Model 2	1	0.93 (0.64, 1.36) P = .71	1.03 (0.70, 1.52) P = .87	1.05 (0.90, 1.23) P = .56
Model 3	1	0.93 (0.62, 1.38) P = .70	1.07 (0.71, 1.61) P = .74	1.07 (0.91, 1.27) P = .42
**Frailty**				
Model 1	1	1.02 (0.53, 1.94) P = .96	0.92 (0.48, 1.77) P = .81	0.96 (0.74, 1.25) P = .76
Model 2	1	0.83 (0.39, 1.76) P = .63	1.13 (0.53, 2.40) P = .76	1.03 (0.76, 1.42) P = .84
Model 3	1	0.67 (0.28, 1.60) P = .37	1.15 (0.48, 2.77) P = .75	1.01 (0.69, 1.46) P = .98

*Notes*: Model 1 unadjusted (n = 785 for total, 350 robust, 370 pre-frail, and 65 frail); Model 2 adjusted by sociodemographic and lifestyle factors (age (continuous), BMI (continuous), smoking status (nonsmoker v. ex-smoker v. current smoker), energy intake (continuous), and supplement use including vitamins, minerals, and/or fish oil (yes v. no)) (n = 775 for total, 344 robust, 368 pre-frail, and 63 frail); Model 3 adjusted by Model 2 plus health (haemoglobin (continuous), number of medications (continuous), number of comorbidities (continuous), and self-rated health (very poor/poor/fair v. good/excellent)) (n = 749 for total, 337 robust, 356 pre-frail, and 56 frail).

a Bottom tertile ≤−0.47, n = 262; middle tertile −0.46 to 0.17, n = 262; top tertile ≥0.18, n = 261.

b Bottom tertile ≤−0.48, n = 262; middle tertile −0.47 to 0.31, n = 262; top tertile ≥0.32, n = 261.

c Bottom tertile ≤−0.41, n = 262; middle tertile −0.40 to 0.41, n = 262; top tertile ≥0.42, n = 261.

The ‘discretionary-starchy vegetables-processed meats’ dietary pattern score as both categorical and continuous variables was not associated with the prevalence of frailty in unadjusted and fully adjusted analyses. Compared to the reference bottom tertile, the top tertile of the ‘discretionary-starchy vegetables-processed meats’ dietary pattern score was not associated with pre-frailty in unadjusted analyses, but was associated with increased prevalence of pre-frailty in fully adjusted analyses (OR 1.75 [95% CI: 1.08, 2.83, P = .022]).

The ‘wholegrains-milk-other fruits’ dietary pattern score as both categorical and continuous variables was not associated with the prevalence of pre-frailty and frailty in unadjusted and fully adjusted analyses.

### 3.4 Longitudinal analyses of associations between dietary patterns, incident pre-frailty and frailty from robust

For the longitudinal analyses from robust, 296 of the 350 men who were robust at baseline nutrition had data available at 3-year follow-up (see Fig. 1). Of the 296 men, the incidence of pre-frailty was 52.4% (n = 155) for those who deteriorated from robust to pre-frail, the incidence of frailty was 5.4% (n = 16) for those who deteriorated from robust to frail, and 42.2% (n = 125) remained robust. The median daily dietary pattern score according to transitions in frailty status from robust at baseline nutrition are available in Supplementary Table 3.

Longitudinal analyses evaluating associations between dietary pattern scores, incident pre-frailty and frailty from robust are presented in Table 3. The ‘vegetables-legumes-seafood’ and ‘discretionary-starchy vegetables-processed meats’ dietary pattern scores as both categorical and continuous variables were not associated with the incidence of pre-frailty and frailty in unadjusted and fully adjusted analyses. However, in fully adjusted analyses the middle tertile of the ‘vegetables-legumes-seafood’ dietary pattern score had a non-significant trend towards reduced incident pre-frailty (OR 0.52 [95% CI: 0.27, 1.00, P = .050]). A higher ‘wholegrains-milk-other fruits’ dietary pattern score was associated with reduced incidence of pre-frailty and frailty in unadjusted analyses (OR 0.76 [95% CI: 0.60, 0.97, P = .028] and OR 0.60 [95% CI: 0.39, 0.93, P = .023]) but was not associated in fully adjusted analyses. The ‘wholegrains-milk-other fruits’ dietary pattern score as a categorical variable was not associated with the incidence of pre-frailty and frailty in unadjusted and fully adjusted analyses.

**Table 3:** Longitudinal associations between dietary pattern factor scores, incident pre-frailty and frailty from robust using multinomial logistic regression presented as odds ratios (n = 296).

Dietary Pattern Factor Scores	Bottom tertile (reference category)	Middle tertile	Top tertile	As continuous variable (per 1 increment)
Factor 1: ‘Vegetables-legumes-seafood’^a^				
**Pre-frailty**				
Model 1	1	0.61 (0.33, 1.13) P = .12	0.58 (0.32, 1.03) P = .063	0.82 (0.64, 1.03) P = .090
Model 2	1	0.51 (0.27, 0.97) P = .041	0.60 (0.32, 1.11) P = .10	0.86 (0.67, 1.11) P = .26
Model 3	1	0.52 (0.27, 1.00) P = .050	0.65 (0.35, 1.24) P = .20	0.91 (0.70, 1.18) P = .47
**Frailty**				
Model 1	1	0.88 (0.25, 3.15) P = .84	0.56 (0.15, 2.07) P = .38	1.12 (0.77, 1.63) P = .55
Model 2	1	0.61 (0.15, 2.44) P = .48	0.51 (0.12, 2.25) P = .38	1.06 (0.69, 1.61) P = .80
Model 3	1	0.85 (0.18, 4.03) P = .83	0.50 (0.10, 2.55) P = .40	0.97 (0.62, 1.52) P = .89
Factor 2: ‘Discretionary-starchy vegetables-processed meats’^b^				
**Pre-frailty**				
Model 1	1	0.85 (0.48, 1.50) P = .57	0.98 (0.55, 1.74) P = .94	0.93 (0.73, 1.19) P = .55
Model 2	1	1.16 (0.62, 2.16) P = .64	1.88 (0.89, 3.98) P = .097	1.26 (0.91, 1.75) P = .17
Model 3	1	1.19 (0.63, 2.26) P = .59	1.84 (0.86, 3.92) P = .12	1.22 (0.87, 1.71) P = .25
**Frailty**				
Model 1	1	1.79 (0.49, 6.56) P = .38	1.45 (0.36, 5.78) P = .60	0.74 (0.41, 1.34) P = .32
Model 2	1	1.97 (0.47, 8.26) P = .35	1.41 (0.25, 7.88) P = .70	0.68 (0.32, 1.47) P = .33
Model 3	1	1.82 (0.36, 9.25) P = .47	1.94 (0.30, 12.64) P = .49	0.73 (0.33, 1.63) P = .44
Factor 3: ‘Wholegrains-milk-other fruits’^c^				
**Pre-frailty**				
Model 1	1	1.34 (0.74, 2.42) P = .34	0.66 (0.37, 1.16) P = .15	0.76 (0.60, 0.97) P = .028
Model 2	1	1.29 (0.70, 2.40) P = .42	0.80 (0.43, 1.45) P = .45	0.84 (0.64, 1.09) P = .19
Model 3	1	1.25 (0.66, 2.35) P = .49	0.82 (0.44, 1.54) P = .54	0.84 (0.63, 1.10) P = .20
**Frailty**				
Model 1	1	1.09 (0.31, 3.91) P = .89	0.69 (0.20, 2.41) P = .56	0.60 (0.39, 0.93) P = .023
Model 2	1	0.82 (0.20, 3.34) P = .79	0.75 (0.19, 3.00) P = .68	0.67 (0.41, 1.10) P = .11
Model 3	1	0.96 (0.23, 4.06) P = .96	0.59 (0.13, 2.67) P = .49	0.70 (0.42, 1.17) P = .17

*Notes*: Model 1 unadjusted (n = 296 for total, 125 robust, 155 pre-frail, and 16 frail); Model 2 adjusted by sociodemographic and lifestyle factors (age (continuous), BMI (continuous), smoking status (nonsmoker v. ex-smoker v. current smoker), energy intake (continuous), and supplement use including vitamins, minerals and/or fish oil (yes v. no)) (n = 293 for total, 125 robust, 152 pre-frail, and 16 frail); Model 3 adjusted by Model 2 plus health (haemoglobin (continuous), number of medications (continuous), number of comorbidities (continuous), and self-rated health (very poor/poor/fair v. good/excellent)) (n = 286 for total, 124 robust, 147 pre-frail, and 15 frail).

a Bottom tertile ≤−0.47, n = 89; middle tertile −0.46 to 0.17, n = 92; top tertile ≥0.18, n = 115.

b Bottom tertile ≤−0.48, n = 106; middle tertile −0.47 to 0.31, n = 98; top tertile ≥0.32, n = 92.

c Bottom tertile ≤−0.41, n = 103; middle tertile −0.40 to 0.41, n = 93; top tertile ≥0.42, n = 100.

### 3.5 Longitudinal subanalyses of associations between dietary patterns, incident robust and frailty from pre-frail

For the longitudinal subanalyses from pre-frail, 273 of the 370 men who were pre-frail at baseline nutrition had data available at 3-year follow-up (see Fig. 1). Of the 273 men, the incidence of frailty was 26.7% for those who deteriorated from pre-frail to frail (n = 73), 65.2% remained pre-frail (n = 178), and incidence of robust was 8.1% (n = 22) for those who improved from pre-frail to robust. The median daily dietary pattern score according to transitions in frailty status from pre-frail at baseline nutrition are available in Supplementary Table 4. Longitudinal subanalyses evaluating associations between dietary pattern scores, incident robust and incident frailty are presented in Supplementary Table 5. There were no associations between dietary pattern scores as both continuous and categorical variables with incident robust and incident frailty from pre-frail.

## 4 Discussion

To the authors’ knowledge, this is the first study to investigate cross-sectional and longitudinal associations between empirically derived dietary patterns and frailty in older men aged 75 years and over. We found that the top tertile and a higher ‘vegetables-legumes-seafood’ dietary pattern score were associated with reduced prevalence of frailty, whilst the top tertile of the ‘discretionary-starchy vegetables-processed meats’ dietary pattern score was associated with increased prevalence of pre-frailty.

Similar to our results, a systematic review and meta-analysis, involving six cohort and cross-sectional studies in a subgroup analysis, five using the *a priori* approach and one using a hybrid approach, found that healthy dietary patterns with high intakes of fruits, vegetables, wholegrains, and fish, and low intakes of high-fat dairy products, red meats, processed meats, and refined grains were associated with reduced risks of frailty in older adults aged 65 years and over [6]. Another systematic review and meta-analysis of four longitudinal studies involving *a priori* diet quality indices found that higher adherence to the Mediterranean dietary pattern characterised by high consumption of fruits, vegetables, legumes, nuts, cereals, fish, and olive oil, and low consumption of meats and dairy, were associated with reduced incident frailty with a follow-up range of 2–6 years in adults aged 60 years and over [24]. Other *a priori* approaches measuring adherence to healthy dietary patterns based on dietary guidelines have been associated with frailty, including: the revised Dietary Guidelines Index-2013 cross-sectionally associated with reduced prevalence of frailty in older men aged 75 years and over from this CHAMP cohort [8]; the Alternative Healthy Eating Index cross-sectionally associated with reduced prevalence of pre-frailty and frailty in men aged 60 years and over in the United States Physicians’ Health Study [25]; and the Diet Quality Index Revised associated with reduced prevalence of frailty in cross-sectional analyses, and reduced incidence of frailty over 4.6 years in longitudinal analyses of older men aged 65 years and over in the United States Osteoporotic Fractures in Men Study [26].

There are a limited number of studies that have investigated the relationship between *a posteriori* empirically derived dietary patterns and frailty, which also yielded similar results: the ‘vegetables-oils-fish’ dietary pattern was associated with reduced prevalence of frailty, whilst the ‘white rice and salted vegetables’ dietary pattern was associated with increased prevalence of frailty, and the ‘noodles and meat’ dietary pattern was associated with increased prevalence of pre-frailty and frailty in cross-sectional analyses of older adults aged 65 years and over in Korea [27]; the ‘prudent’ dietary pattern high in poultry, fish, vegetables, legumes, pasta, rice, and eggs was associated with reduced incident frailty, whilst the ‘high fat/low fibre’ dietary pattern was associated with increased incident frailty over 3 years in older men aged 70 years and over in the United Kingdom [28]; the ‘protein-rich’ dietary pattern consisting of meat and fish was negatively associated, whilst the ‘salt and pickles’ dietary pattern consisting of salty foods, soup dishes, and seasoning, and ‘sugar and fat’ dietary pattern consisting of fats, oils, bread, confectionery, and sugar was positively associated with frailty over 3 years in adults aged 60 years and over in Japan [29]; compared to the ‘healthy’ dietary pattern high in fruits and fish, the ‘pasta’ dietary pattern high in starchy food including pasta, rice, potatoes, and bread was associated with increased incident frailty, and additionally the ‘biscuits and snacking’ dietary pattern high in biscuits and cakes, and low in fruits, vegetables, and fish, and the ‘pasta’ dietary pattern were associated with increased risk of muscle weakness over 12 years in older men aged 65 years and over in France [30]; and the ‘prudent’ dietary pattern with high consumption of olive oil, vegetables, potatoes, legumes, fatty fish, and pasta, was associated with reduced incident frailty over 3.5 years, and additionally the ‘Westernised’ dietary pattern with high consumption of refined bread, full-fat dairy, French fries, red meats, processed meats, sugar and honey, and low intakes of wholegrains, fruit, low-fat dairy, and vegetables was associated with increased risks of slow walking speed and weight loss over 3.5 years in adults aged 60 years and over in Spain [31].

There are several mechanisms that could explain why the ‘vegetables-legumes-seafood’ dietary pattern has potential to be protective against frailty. Firstly, similar healthy dietary patterns have shown to improve cardiometabolic health in older adults [32,33], which have a bidirectional relationship with frailty [34]. Vegetable and fruit intake has also been inversely associated with incident frailty in a systematic review and meta-analysis of seven cohort studies with a follow-up range of 2–20 years in adults aged 35 years and over [35]. In particular, the’ vegetables-legumes-seafood’ dietary pattern in the present study consists of a variety of vegetables, legumes, and fruits, and thus contains different phytochemicals that have antioxidant properties such as carotenoids and flavonoids [36,37]. It has been previously shown through the CHAMP cohort that poor dietary antioxidant intake was associated with increased incident frailty over 3 years in older men aged 75 years and over [38]. Antioxidants reduce oxidative stress which can accelerate muscle breakdown and decrease muscle growth, and hence reduce musculoskeletal system damage to prevent reduction in physical capacity and impact on frailty components [39]. Frailty has also been cross-sectionally associated with elevated levels of IL-6 in older men aged 75 years and over from the CHAMP cohort [40], and diets with higher inflammatory potential have been associated with increased incident frailty over 8 years in adults aged 45 years and over [41]. Carotenoids and flavonoids also have anti-inflammatory properties [36,42].

Seafood, in particular fatty fish, contain omega-3 polyunsaturated fatty acids with anti-inflammatory actions that may be protective of muscle function [43]. Higher fatty fish consumption was cross-sectionally associated with increased grip strength in adults aged 59–73 years [43]. Lower dietary eicosapentaenoic acid and docosahexaenoic acid intake was cross-sectionally associated with reduced motor performance in older men aged 85 years and over [44]. Fatty fish, dietary eicosapentaenoic acid and docosahexaenoic acid were also associated with reduced functional impairments over 6 years, and reduced incident muscle weakness over 5 years in adults aged 60 years and over [45,46]. Furthermore, seafood, legumes, and nuts and seeds provide a source of protein that is important due to higher protein requirements in old age to prevent sarcopenia, which is closely related to frailty [47,48]. Dietary leucine intake was associated with reduced incident frailty and reduced risks of impaired lower extremity function, whilst fatty and white fish consumption were also associated with reduced risks of impaired lower extremity function over 7 years in adults aged 60 years and over [49]. In a recent study, the replacement of red meat with seafood and legumes was associated with reduced incidence of frailty over 22 years in women aged 60 years and over [47]. Similarly, fish and fish protein intake was cross-sectionally associated with reduced falls whilst red meat intake was associated with increased falls in older men aged 83 years and over [50], whereby frailty is a predictor of falls [51].

In the current study, empirically derived dietary patterns were not longitudinally associated with incident pre-frailty and frailty. Frailty has been associated with poor oral health in older men from the CHAMP cohort [52], and compared to those with natural functional tooth units only, older men without natural functional tooth units had nutritionally inadequate intakes of fibre [53]. Thus, an explanation for the observed cross-sectional associations could be that those with frailty tend to avoid fibrous foods present in the ‘vegetables-legumes-seafood’ dietary pattern that may be hard to chew or stringy. Older men also tend to have limited meal preparation skills [54], and those with frailty may not know how to prepare foods included in the ‘vegetables-legumes-seafood’ dietary pattern which are mostly unprocessed. In the present study, the top tertile of the ‘discretionary-starchy vegetables-processed meats’ dietary pattern that contained more processed foods was also associated with increased prevalence of pre-frailty. In other cross-sectional studies, consuming more unprocessed or minimally processed foods was inversely associated with frailty in older adults aged 65 years and over [55], and higher ultra-processed food consumption was also associated with increased prevalence of pre-frailty and frailty in adults aged 60 years and over [56].

There are several study limitations. As our study is observational, residual confounding cannot be ruled out and causation cannot be determined. However, it was possible to investigate the temporal direction of the relationships between empirically derived dietary patterns with pre-frailty and frailty from cross-sectional and longitudinal analyses. Bias due to loss to follow-up could occur when those who are less healthy become frail and drop out of the study or died prior to follow-up assessment. We have no follow-up data so are unable to investigate whether there were differential losses according to exposures and outcomes between participants and non-participants. The period of follow-up over 3 years was relatively short, and as we excluded those who were pre-frail or frail at baseline, the number of participants and number of events for transitions in frailty status reduced in longitudinal analyses. Dietary exposure and other measures were only from a single timepoint that may have changed over the follow-up period, possibly resulting in misclassification of exposure factors to some extent but which would be expected to nullify rather than create positive findings. This could also potentially explain the lack of significant associations between empirically derived dietary patterns with incident pre-frailty and frailty in longitudinal analyses. Food groupings were obtained using FoodWorks which is based on the Australian Guide to Healthy Eating, and we used an adapted definition for a serve of discretionary which included sugar, solid fats, and alcohol. In factor analysis, there is potential subjectivity in the number of factors retained as they are based on empirical guidelines rather than exact quantitative solutions, in the labelling of the dietary patterns and the groupings of food items [5,21,57]. However, the food groups used in the present study were based on those converted from FoodWorks. The three dietary patterns derived explained 23.7% of the total variance suggests there is heterogeneity amongst dietary intake in this cohort of older men and the potential existence of other dietary patterns. However, it is similar to the total variance of prior studies [27,57], and the ‘percentage of variance explained’ should be interpreted with caution as it is a function of the number of food group items included in the factor analysis: as food is more broadly classified with a lower number of food groups, the greater the total variance explained by resultant dietary patterns [58]. The current study focused on the frailty outcome defined by the Fried frailty phenotype criteria [14], other measures of physical frailty such as the Rockwood Frailty Index [59], and cognitive frailty which combines physical frailty and cognitive impairment [60] could be considered in the future. Our study was limited to community-dwelling men and the dietary patterns were derived from this study population, therefore our results may not apply to older women or institutionalised populations.

The strength of our study is that we explored the relationships of both cross-sectional associations between empirically derived dietary patterns with the prevalence of pre-frailty and frailty, and longitudinal associations between empirically derived dietary patterns with incident pre-frailty and frailty over time from robust. Additionally, we conducted subanalyses on the longitudinal associations between empirically derived dietary patterns with incident robust and frailty over time from pre-frail. Examining empirically derived dietary patterns as exposure rather than diet quality indices, allows for the exploration of dietary patterns independent of prior knowledge [5]. We used a validated dietitian-administered diet history questionnaire, which has been indicated for older adults due to the non-reliance on short-term memory and low respondent burden [9]. We considered multiple confounders for inclusion in the model, and adjustments included supplement use including vitamins, minerals, and/or fish oil, medications, and comorbidities that could impact on the development of frailty and dietary intake, and energy intake that would also account for under or over-reporting. A further strength of this study is that the participants were from CHAMP that included a representative group of older Australian men [7].

## 5 Conclusion

The top tertile and higher adherence to the ‘vegetables-legumes-seafood’ dietary pattern were cross-sectionally associated with reduced prevalence of frailty, whilst the top tertile of the ‘discretionary-starchy vegetables-processed meats’ dietary pattern was cross-sectionally associated with an increased prevalence of pre-frailty. Consumption of a ‘vegetables-legumes-seafood’ dietary pattern appears to be less favoured by frail older men.

## Conflict of interest

The authors declare no conflicts of interest.

## Funding

The CHAMP study is funded by the 10.13039/501100000925National Health and Medical Research Council (project grant no. 301916), Ageing and Alzheimers Research Institute, Ageing and Alzheimers Research Foundation, and the 10.13039/501100007811Sydney Medical School Foundation. This research was supported by the Australian Research Council Centre of Excellence in Population Ageing Research (project number CE170100005). RL was supported by an Australian Government Research Training Program Scholarship and an Australian Research Council Centre of Excellence in Population Aging Research Scholarship. RVR was supported by a Charles Perkins Centre Early Career Fellowship from Jennie Mackenzie. The funders were not involved in the conception, design, performance, or approval of this work.

## Author contributions

R.L., R.R., V.N., and V.H. conceptualised the analyses presented in this manuscript. R.L., R.R., and V.H. designed the analyses. R.L. performed the data analyses. R.L., R.R., and V.H. interpreted the data. R.L. wrote the manuscript. V.N., F.M.B., L.M.W., D.J.H., D.G.L.C., M.J.S., and V.H. were principal investigators in the original and succeeding waves of the Concord Health and Ageing in Men Project, each contributing to design of the Project, data collection, and analysis. All authors played a critical role in reviewing, editing and approved the final version of the manuscript. R.L. and V.H. had full access to all of the data in the study and take responsibility for the integrity of the data and the accuracy of the data analysis.

## Ethical standards

The CHAMP study was conducted according to the guidelines laid down in the Declaration of Helsinki and all procedures involving human subjects were approved by the Concord Hospital Human Research Ethics Committee (HREC/14/CRGH/17). Written informed consent was obtained from all participants.
