# Supplementary material for: Cross-sectional and longitudinal associations between empirically derived dietary patterns and frailty among older men: The Concord Health and Ageing in Men Project
Source: J Nutr Health Aging. 2024 Jan 1;28(1):100021. doi: 10.1016/j.jnha.2023.100021 (PMC12877762; doi:10.1016/j.jnha.2023.100021)
Supplement: Supplementary file 1 [file mmc1.pdf]

I. **Supplementary Figure 1.** Scree plot for the factor analysis

II. **Supplementary Table 1.** Factor loadings with varimax rotation to determine the association between 23 food groups and factors representing dietary patterns among older Australian men (n = 785)

III. **Supplementary Table 2.** Dietary intake (median and interquartile range) according to dietary pattern factor score tertiles (n = 785)

IV. **Supplementary Table 3.** Median (interquartile range) daily dietary pattern scores and according to transitions in frailty status from robust at baseline (n = 296)

V. **Supplementary Table 4.** Median (interquartile range) daily dietary pattern scores and according to transitions in frailty status from pre-frail at baseline (n = 273)

VI. **Supplementary Table 5.** Longitudinal associations between dietary pattern factor scores, incident robust and incident frailty from pre-frail using multinomial logistic regression presented as odds ratios (n = 273)

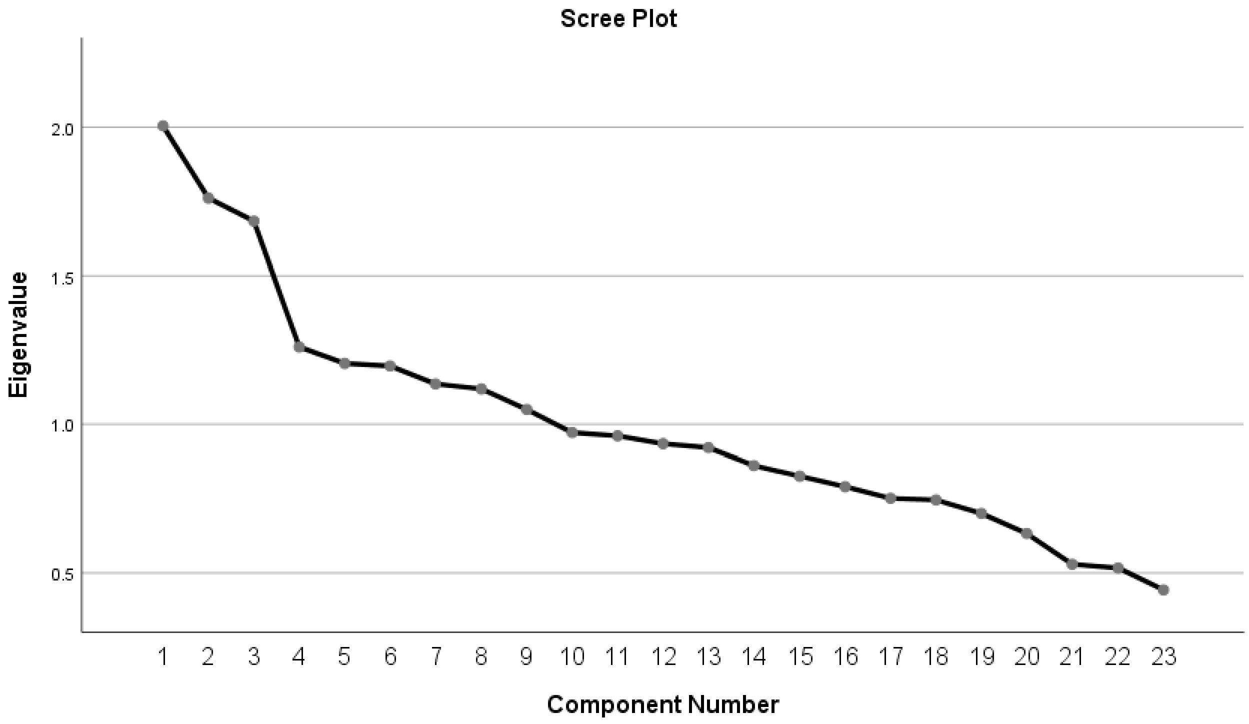

Supplementary Figure 1. Scree plot for the factor analysis

Supplementary Table 1. Factor loadings with varimax rotation to determine the association between 23 food groups and factors representing dietary patterns among older Australian men (n = 785)

**Foods**	**Factor 1: ‘Vegetables-legumes-seafood’**	**Factor 2: ‘Discretionary-starchy vegetables-processed meats’**	**Factor 3: ‘Wholegrains-milk-other fruits’**
Other vegetables	**0.73**	0.29	0.07
Dark green vegetables	**0.62**	-0.05	-0.08
Red orange vegetables	**0.60**	0.10	0.17
Legumes	**0.43**	-0.05	-0.04
Seafood	**0.31**	-0.06	0.01
Poultry	0.15	-0.10	0.10
Discretionary	-0.22	**0.71**	0.05
Starchy vegetables	0.27	**0.54**	0.08
Processed meats	-0.14	**0.52**	0.04
Fruit juice	0.01	**0.38**	-0.03
Eggs	0.11	**0.32**	0.04
Red meats	0.17	**0.31**	-0.15
Cheese	-0.04	0.27	-0.12
Milk alternatives	0.17	-0.17	0.16
Organ meats	-0.05	0.13	-0.02
Wholegrains	-0.02	0.00	**0.73**
Refined grains	0.13	0.11	**-0.66**
Milk	-0.19	0.20	**0.41**
Yoghurt	0.06	-0.08	**0.37**
Soy products	0.26	-0.19	**-0.34**
Other fruits	0.21	0.05	**0.31**
Citrus, melons and berries	0.11	-0.16	0.26
Nuts and seeds	0.09	-0.04	0.18

% variance explained	8.71	7.66	7.32
Eigen value	2.00	1.76	1.68

Factor loadings with absolute value >0.30 and <-0.30 are shown in bold to indicate major contributing food groups and non-contributing food groups for each dietary pattern respectively.

Supplementary Table 2. Dietary intake (median and interquartile range) according to dietary pattern factor score tertiles (n = 785)

		**Factor 1: ‘Vegetables-legumes-seafood’**				**Factor 2: ‘Discretionary-starchy vegetables-processed meats’**				**Factor 3: ‘Wholegrains-milk-other fruits’**			
**Variables**	**All n = 785**	**Bottom n = 262**	**Middle n = 262**	**Top n = 261**	**P value[1]**	**Bottom n = 262**	**Middle n = 262**	**Top n = 261**	**P value[1]**	**Bottom n = 262**	**Middle n = 262**	**Top n = 261**	**P value[1]**
Energy intake (kJ)	8824.6 (7311.1-10515.4)	8465.5 (6913.9-10079.8)[a,b]	8792.5 (7429.8-10332.2)[a,c]	9267.3 (7695.3-10886.1)[b,c]	.66[a] .006[b] .060[c]	7482.2 (6425.2-8742.1)[a,b]	8572.3 (7411.2-9884.4)[a,c]	10569.6 (9219.3-12005.6)[b,c]	<.001[a,b,c]	8402.5 (6811.1-10203.9)[a,b]	8356.3 (7185.6-9956.8)[a,c]	9600.5 (8312.6-11015.5)[b,c]	1.00[a] <.001[b,c]
Nutrients													
Protein (g/kg body weight)	1.3 (1.0-1.6)	93.0 (76.7-110.4)[a,b]	98.1 (84.7-115.9)[a,c]	111.2 (93.3-132.8)[b,c]	.88[a] <.001[b,c]	1.1 (0.9-1.4)[a,b]	1.2 (1.0-1.5)[a,c]	1.5 (1.3-1.8)[b,c]	.016[a] <.001[b,c]	1.3 (1.0-1.6)[a,b]	1.2 (1.0-1.5)[a,c]	1.4 (1.2-1.7)[b,c]	.49[a] <.001[b,c]
Protein (g)	100.1 (84.0-119.4)	93.0 (76.7-110.4)[a,b]	98.1 (84.7-115.9)[a,c]	111.2 (93.3-132.8)[b,c]	.24[a] <.001[b,c]	86.5 (72.4-101.3)[a,b]	97.7 (84.0-113.5)[a,c]	116.5 (101.4-131.4)[b,c]	<.001[a,b,c]	95.3 (77.3-116.1)[a,b]	95.7 (82.9-113.3)[a,c]	109.8 (94.3-125.6)[b,c]	1.00[a] <.001[b,c]
Protein (%E)	19.3 (17.1-22.1)	18.7 (16.6-21.1)[a,b]	19.1 (17.0-21.6)[a,c]	20.5 (17.5-23.1)[b,c]	1.00[a] .003[b] .030[c]	20.4 (17.3-23.0)[a,b]	19.2 (16.8-22.1)[a,c]	18.7 (17.0-21.0)[b,c]	.043[a] <.001[b] 1.00[c]	19.6 (16.9-22.8)	18.9 (17.0-21.5)	19.4 (17.3-22.1)	.28

Carbohydrate (g)	201.0 (163.7-242.8)	196.4 (154.9-241.2)	198.5 (163.4-236.9)	211.6 (170.8-257.1)	.15	172.6 (145.3-209.3)[a,b]	193.1 (168.8-235.7)[a,c]	240.6 (200.8-283.0)[b,c]	.001[a] <.001[b,c]	182.6 (148.7-228.9)[a,b]	185.8 (156.6-226.3)[a,c]	233.8 (193.1-267.9)[b,c]	1.00[a] <.001[b,c]
Carbohydrate (%E)	37.6 (31.9-42.5)	38.1 (32.2-42.8)	37.2 (31.2-42.0)	37.4 (32.4-42.5)	.29	38.8 (31.6-43.6)	37.1 (31.9-42.2)	37.2 (32.3-41.5)	.072	36.3 (30.1-42.2)[a,b]	36.4 (31.6-41.5)[a,c]	39.9 (34.9-43.2)[b,c]	1.00[a] <.001[b,c]
Added sugar (g)	30.8 (14.4-50.5)	37.2 (21.6-55.6)[a,b]	30.2 (15.7-51.6)[a,c]	24.9 (10.9-40.9)[b,c]	.026[a] <.001[b] .061[c]	17.7 (9.4-31.4)[a,b]	31.1 (16.5-46.9)[a,c]	51.9 (27.8-73.4)[b,c]	<.001[a,b,c]	27.5 (13.0-48.2)[a,b]	28.3 (15.2-48.9)[a,c]	33.7 (18.3-55.0)[b,c]	1.00[a] .018[b] .030[c]
Added sugar (%E)	5.7 (3.0-9.2)	7.8 (4.5-10.8)[a,b]	5.7 (3.2-9.0)[a,c]	4.4 (2.1-7.5)[b,c]	<.001[a,b] .013[c]	3.9 (2.0-6.7)[a,b]	5.8 (3.3-8.8)[a,c]	8.3 (4.6-11.3)[b,c]	<.001[a,b,c]	5.4 (2.8-9.2)	5.6 (3.2-9.5)	6.5 (3.2-8.9)	.12
Dietary fibre (g)	26.3 (21.1-32.7)	21.4 (16.5-26.1)[a,b]	25.9 (22.2-31.3)[a,c]	32.1 (27.1-40.7)[b,c]	<.001[a,b,c]	25.2 (19.6-31.2)[a,b]	25.8 (20.6-31.3)[a,c]	28.3 (22.4-35.7)[b,c]	1.00[a] .003[b] .010[c]	21.6 (17.1-27.3)[a,b]	26.1 (21.5-31.1)[a,c]	31.1 (25.9-38.2)[b,c]	<.001[a,b,c]
Dietary fibre (%E)	2.4 (2.0-3.0)	21.4 (16.5-26.1)[a,b]	25.9 (22.2-31.3)[a,c]	32.1 (27.1-40.7)[b,c]	<.001[a,b,c]	2.8 (2.1-3.3)[a,b]	2.4 (2.0-2.9)[a,c]	2.1 (1.8-2.7)[b,c]	<.001[a,b] .001[c]	2.1 (1.7-2.6)[a,b]	2.4 (2.0-3.0)[a,c]	2.7 (2.2-3.2)[b,c]	<.001[a,b] .002[c]
Total fat (g)	83.6 (63.4-108.3)	80.7 (61.5-107.3)	84.8 (63.3-107.4)	84.6 (63.9-109.5)	.66	67.7 (50.6-88.9)[a,b]	83.3 (62.7-104.1)[a,c]	102.2 (80.0-126.8)[b,c]	<.001[a,b,c]	80.5 (59.8-108.3)	83.8 (63.6-105.7)	86.9 (64.6-111.7)	.23

Total fat (%E)	35.2 (29.8-40.8)	35.9 (29.9-42.2)[a,b]	35.6 (30.4-41.4)[a,c]	33.8 (29.2-39.0)[b,c]	1.00[a] .008[b] .038[c]	33.7 (28.3-40.0)[a,b]	35.2 (29.6-41.4)[a,c]	36.1 (31.8-40.5)[b,c]	.16[a] .003[b] .94[c]	35.2 (29.9-42.1)[a,b]	36.2 (30.8-41.8)[a,c]	33.7 (28.4-38.7)[b,c]	.24[a] .061[b] .004[c]
Saturated fat (g)	28.3 (21.0-37.0)	30.7 (22.6-39.5)[a,b]	28.8 (21.1-37.7)[a,c]	25.5 (19.8-33.9)[b,c]	.66[a] <.001[b] .008[c]	21.0 (17.2-26.9)[a,b]	28.4 (22.4-34.6)[a,c]	37.5 (29.4-46.2)[b,c]	<.001[a,b,c]	26.4 (19.8-35.1)	29.0 (21.4-35.9)	29.2 (21.6-39.3)	.039[†]
Saturated fat (%E)	11.9 (9.7-14.4)	13.5 (11.1-15.4)[a,b]	12.1 (10.1-14.4)[a,c]	10.4 (8.5-12.2)[b,c]	.005[a] <.001[b,c]	10.6 (8.6-12.3)[a,b]	12.1 (9.9-14.6)[a,c]	13.1 (11.2-15.6)[b,c]	<.001[a,b] .049[c]	11.6 (9.6-14.2)[a,b]	12.4 (10.3-14.9)[a,c]	11.4 (9.5-13.7)[b,c]	.069[a] 1.00[b] .023[c]
Monounsaturated fat (g)	34.3 (25.0-47.3)	31.5 (23.5-43.2)[a,b]	33.6 (25.5-47.2)[a,c]	37.6 (25.9-49.9)[b,c]	.88[a] .010[b] .12[c]	28.3 (19.8-39.4)[a,b]	32.9 (23.7-45.1)[a,c]	40.0 (30.3-52.3)[b,c]	.069[a] <.001[b] .003[c]	34.1 (23.5-49.7)	34.3 (25.9-47.3)	33.8 (25.1-46.4)	1.00
Polyunsaturated fat (g)	12.4 (8.6-16.7)	11.1 (7.5-15.5)[a,b]	12.0 (8.5-16.6)[a,c]	13.6 (10.1-18.3)[b,c]	1.00[a] <.001[b] .18[c]	11.3 (7.3-15.9)[a,b]	11.7 (8.6-16.3)[a,c]	13.6 (10.3-17.9)[b,c]	1.00[a] .001[b] <.001[c]	11.3 (7.5-15.7)[a,b]	11.7 (8.3-16.8)[a,c]	13.3 (9.4-17.6)[b,c]	1.00[a] <.001[b] .12[c]
Linoleic acid (g)	9.8 (6.8-13.7)	8.8 (6.0-12.5)[a,b]	9.5 (6.7-13.8)[a,c]	11.0 (7.6-15.0)[b,c]	.88[a] <.001[b] .049[c]	9.2 (5.6-13.3)[a,b]	9.2 (6.8-13.4)[a,c]	11.0 (8.0-14.8)[b,c]	1.00[a] .003[b] .001[c]	9.2 (5.9-13.0)[a,b]	9.5 (6.7-13.8)[a,c]	10.9 (7.6-14.6)[b,c]	1.00[a] <.001[b] .12[c]

Linoleic acid (%E)	4.1 (3.2-5.4)	3.9 (3.0-5.1)[a,b]	4.1 (3.1-5.3)[a,c]	4.4 (3.4-5.7)[b,c]	.88[a] .030[b] .94[c]	4.5 (3.4-6.0)[a,b]	4.1 (3.3-5.3)[a,c]	3.9 (3.0-5.0)[b,c]	.11 .023 .45	3.8 (3.0-5.2)[a,b]	4.3 (3.3-5.5)[a,c]	4.3 (3.2-5.6)[b,c]	.005[a] .010[b] 1.00[c]
Alpha-linolenic acid (g)	1.3 (0.9-1.9)	1.3 (0.9-2.0)	1.2 (0.9-1.8)	1.3 (0.9-1.9)	.13	1.1 (0.7-1.7)[a,b]	1.3 (0.9-1.9)[a,c]	1.6 (1.1-2.1)[b,c]	.001[a] <.001[b,c]	1.2 (0.8-1.8)[a,b]	1.2 (0.8-1.9)[a,c]	1.4 (1.0-2.1)[b,c]	1.00[a] .049[b] .030[c]
Alpha-linolenic acid (%E)	0.5 (0.4-0.8)	0.6 (0.4-0.8)	0.5 (0.4-0.8)	0.5 (0.4-0.8)	.28	0.5 (0.4-0.8)	0.5 (0.4-0.8)	0.5 (0.4-0.7)	.73	0.5 (0.4-0.8)	0.5 (0.4-0.8)	0.5 (0.4-0.8)	.30
Long chain omega-3 (mg)	476.4 (291.1-727.2)	377.4 (220.7-565.1)[a,b]	496.4 (305.0-729.9)[a,c]	598.3 (372.2-904.7)[b,c]	.003[a] <.001[b] .010[c]	479.4 (277.8-719.6)	440.8 (267.0-687.6)	498.6 (325.6-800.7)	.54	492.2 (289.1-706.1)	429.3 (268.2-708.9)	514.7 (308.9-776.5)	.074
Cholesterol (mg)	296.7 (230.4-380.9)	277.8 (227.3-363.2)[a,b]	302.0 (233.3-374.9)[a,c]	324.5 (238.9-409.8)[b,c]	.35[a] .006[b] .38[c]	239.1 (178.1-290.8)[a,b]	291.3 (233.5-355.7)[a,c]	387.5 (310.8-481.7)[b,c]	<.001[a,b,c]	288.6 (229.3-379.7)	286.6 (224.8-378.4)	314.4 (244.0-388.3)	.15
Alcohol (g)	4.9 (0.0-15.7)	3.3 (0.0-14.3)	7.5 (0.0-16.4)	5.2 (0.0-17.7)	.057	3.3 (0.0-11.9)[a,b]	5.4 (0.0-16.7)[a,c]	8.5 (0.0-19.1)[b,c]	.35[a] .018[b] .71[c]	7.0 (0.0-18.8)	5.2 (0.0-15.0)	3.4 (0.0-12.5)	.37
Alcohol (%E)	1.8 (0.0-5.2)	1.1 (0.0-4.6)	2.2 (0.0-5.6)	2.0 (0.0-5.2)	.072	1.2 (0.0-4.6)	1.9 (0.0-5.4)	2.1 (0.0-5.2)	.16	2.3 (0.0-6.3)	2.0 (0.0-5.2)	1.1 (0.0-4.0)	.082

Thiamin (mg)	1.7 (1.2-2.2)	1.6 (1.2-2.1)	1.6 (1.2-2.1)	1.8 (1.3-2.3)	.062	1.5 (1.1-1.9)[a,b]	1.7 (1.3-2.1)[a,c]	1.8 (1.4-2.3)[b,c]	.016[a] <.001[b] .53[c]	1.4 (1.1-1.8)[a,b]	1.6 (1.2-2.0)[a,c]	2.1 (1.6-2.6)[b,c]	.001[a] <.001[b,c]
Riboflavin (mg)	2.2 (1.7-2.8)	2.2 (1.7-2.9)	2.1 (1.6-2.6)	2.2 (1.7-3.0)	.38	1.9 (1.4-2.5)[a,b]	2.1 (1.8-2.7)[a,c]	2.5 (1.9-3.3)[b,c]	.005[a] <.001[b,c]	1.8 (1.3-2.2)[a,b]	2.1 (1.7-2.7)[a,c]	2.7 (2.2-3.6)[b,c]	<.001[a,b,c]
Niacin (mg)	50.2 (42.5-61.1)	46.5 (39.0-56.8)[a,b]	49.8 (43.2-59.9)[a,c]	54.8 (45.2-67.2)[b,c]	.016[a] <.001[b] .002[c]	44.8 (38.2-52.8)[a,b]	49.3 (42.0-58.2)[a,c]	58.4 (49.4-68.1)[b,c]	.001[a] <.001[b,c]	46.2 (38.4-56.6)[a,b]	49.0 (40.8-58.1)[a,c]	56.8 (49.0-67.6)[b,c]	.069[a] <.001[b,c]
Vitamin C (mg)	107.1 (74.2-156.2)	75.4 (54.3-109.7)[a,b]	104.3 (80.5-144.3)[a,c]	148.6 (105.6-200.3)[b,c]	<.001[a,b,c]	102.4 (71.4-150.7)	107.0 (73.8-156.5)	113.5 (80.9-165.7)	.35	90.3 (59.0-125.6)[a,b]	112.0 (76.0-155.1)[a,c]	131.7 (89.8-186.1)[b,c]	<.001[a,b] .003[c]
Vitamin D (ug)	4.5 (3.3-6.1)	4.4 (3.2-5.9)	4.5 (3.3-6.1)	5.0 (3.5-6.4)	.070	3.9 (2.8-5.2)[a,b]	4.4 (3.3-6.0)[a,c]	5.3 (4.2-6.8)[b,c]	.069[a] <.001[b,c]	4.5 (3.2-5.8)	4.4 (3.1-5.9)	5.0 (3.6-6.4)	.12
Vitamin E (mg)	9.8 (7.1-13.5)	8.1 (6.0-11.1)[a,b]	9.8 (7.2-13.4)[a,c]	11.8 (8.9-15.5)[b,c]	.005[a] <.001[b,c]	9.1 (6.5-12.7)[a,b]	9.1 (6.9-13.3)[a,c]	11.1 (8.3-14.6)[b,c]	1.00[a] .001[b] <.001[c]	9.5 (6.1-13.3)	9.8 (7.1-13.7)	9.9 (7.7-13.7)	.89

Folate (ug)	415.8 (308.8-550.0)	381.0 (269.5-506.6)[a,b]	407.1 (304.9-522.5)[a,c]	462.8 (341.1-616.4)[b,c]	.49[a] <.001[b] .002[c]	370.0 (289.9-495.7)[a,b]	396.9 (296.6-519.8)[a,c]	464.1 (361.5-614.8)[b,c]	.35[a] <.001[b,c]	333.3 (257.1-455.1)[a,b]	403.2 (317.8-509.8)[a,c]	509.5 (393.8-663.7)[b,c]	<.001[a,b,c]
Vitamin A (ug)	989.2 (737.0-1365.2)	827.1 (608.7-1074.4)[a,b]	971.1 (745.2-1256.7)[a,c]	1286.6 (934.3-1740.0)[b,c]	<.001[a,b,c]	849.6 (597.0-1132.2)[a,b]	944.2 (743.9-1279.7)[a,c]	1240.5 (907.5-1677.8)[b,c]	.069[a] <.001[b,c]	831.3 (609.7-1166.9)[a,b]	993.8 (766.3-1303.6)[a,c]	1186.2 (899.0-1633.2)[b,c]	<.001[a,b,c]
Sodium (mg)	1979.3 (1536.4-2494.5)	1920.2 (1482.9-2430.0)	1909.6 (1501.6-2471.4)	2050.1 (1615.0-2589.0)	.15	1608.2 (1229.5-2008.3)[a,b]	1940.0 (1590.2-2344.5)[a,c]	2438.9 (2006.5-2920.3)[b,c]	<.001[a,b,c]	1906.1 (1424.5-2551.6)[a,b]	1909.2 (1498.3-2378.7)[a,c]	2081.4 (1687.9-2571.8)[b,c]	1.00[a] .049[b] .077[c]
Potassium (mg)	3341.9 (2793.9-4002.7)	2848.3 (2360.8-3452.1)[a,b]	3272.3 (2837.3-3806.3)[a,c]	3914.5 (3361.3-4753.3)[b,c]	<.001[a,b,c]	3082.5 (2445.8-3659.8)[a,b]	3182.8 (2737.5-3859.3)[a,c]	3804.9 (3303.1-4468.9)[b,c]	1.00[a] <.001[b,c]	2829.9 (2381.9-3449.8)[a,b]	3225.0 (2831.0-3827.2)[a,c]	3941.9 (3410.9-4584.4)[b,c]	<.001[a,b,c]
Magnesium (mg)	355.3 (283.0-436.0)	310.9 (248.1-395.9)[a,b]	345.3 (284.1-425.5)[a,c]	406.1 (335.4-489.2)[b,c]	.043[a] <.001[b,c]	319.9 (257.9-406.8)[a,b]	341.7 (278.9-425.1)[a,c]	400.4 (328.1-467.5)[b,c]	.35[a] <.001[b,c]	282.9 (238.8-351.7)[a,b]	344.9 (291.5-416.3)[a,c]	433.3 (375.4-493.4)[b,c]	<.001[a,b,c]

Calcium (mg)	811.5 (619.1-1047.4)	807.4 (619.6-1049.5)	783.0 (607.5-963.7)	857.2 (625.2-1127.1)	.10	684.9 (531.8-899.5)[a,b]	784.5 (623.1-1002.5)[a,c]	947.8 (758.3-1198.7)[b,c]	.001[a] <.001[b,c]	636.9 (493.2-832.9)[a,b]	788.9 (641.4-994.8)[a,c]	1010.3 (814.1-1259.9)[b,c]	<.001[a,b,c]
Phosphorus (mg)	1597.4 (1311.2-1912.7)	1478.8 (1224.8-1848.3)[a,b]	1572.5 (1296.0-1828.8)[a,c]	1742.6 (1456.9-2073.6)[b,c]	.16[a] <.001[b,c]	1355.7 (1115.8-1647.4)[a,b]	1554.6 (1331.9-1787.1)[a,c]	1879.0 (1610.6-2130.7)[b,c]	.001[a,b,c]	1385.4 (1144.0-1709.2)[a,b]	1528.8 (1299.3-1810.6)[a,c]	1847.7 (1612.8-2164.2)[b,c]	.026[a] <.001[b,c]
Iron (mg)	12.9 (10.5-16.0)	11.8 (9.2-14.8)[a,b]	12.6 (10.4-15.0)[a,c]	14.9 (11.9-18.4)[b,c]	.35[a] <.001[b,c]	11.5 (9.0-14.8)[a,b]	12.4 (10.4-14.9)[a,c]	15.0 (12.5-17.9)[b,c]	.069[a] <.001[b,c]	11.3 (9.2-14.0)[a,b]	12.7 (10.4-15.2)[a,c]	15.1 (12.4-18.5)[b,c]	<.001[a,b,c]
Zinc (mg)	13.3 (11.0-16.7)	12.8 (10.1-15.1)[a,b]	13.3 (10.7-16.2)[a,c]	15.0 (11.9-18.2)[b,c]	.16[a] <.001[b] .030[c]	11.4 (9.4-13.8)[a,b]	13.1 (11.4-15.7)[a,c]	16.2 (13.4-18.9)[b,c]	<.001[a,b,c]	12.4 (9.8-15.9)[a,b]	12.8 (10.7-16.0)[a,c]	14.8 (12.8-17.9)[b,c]	.88[a] <.001[b,c]
Iodine (ug)	110.9 (85.1-146.5)	113.3 (89.3-152.7)	106.4 (78.7-144.3)	113.3 (86.5-144.3)	.48	95.7 (74.4-128.0)[a,b]	107.0 (81.8-144.1)[a,c]	131.2 (101.4-170.0)[b,c]	.005[a] <.001[b,c]	94.9 (72.2-119.2)[a,b]	107.0 (82.1-145.0)[a,c]	137.5 (102.4-178.5)[b,c]	.005[a] <.001[b,c]
Food groups													

Other vegetables (serves/d)	1.3 (0.8-2.1)	0.8 (0.5-1.1)^{a,b}	1.4 (0.9-1.9)^{a,c}	2.1 (1.4-3.2)^{b,c}	<.001^{a,b,c}	1.2 (0.7-1.9)^{a,b}	1.2 (0.8-1.9)^{a,c}	1.6 (0.9-2.4)^{b,c}	.66^{a} <.001^{b,c}	1.2 (0.7-2.0)	1.4 (0.8-2.0)	1.3 (0.9-2.2)	.18
Dark green vegetables (serves/d)	0.2 (0.1-0.5)	0.1 (0.0-0.2)^{a,b}	0.2 (0.1-0.4)^{a,c}	0.5 (0.2-0.8)^{b,c}	<.001^{a,b,c}	0.2 (0.1-0.5)	0.2 (0.1-0.4)	0.2 (0.0-0.4)	.28	0.2 (0.0-0.4)	0.2 (0.1-0.5)	0.2 (0.1-0.5)	.20
Red orange vegetables (serves/d)	0.9 (0.5-1.3)	0.5 (0.3-0.8)^{a,b}	0.9 (0.6-1.2)^{a,c}	1.4 (0.9-2.1)^{b,c}	<.001^{a,b,c}	0.9 (0.4-1.4)	0.8 (0.5-1.2)	0.9 (0.5-1.6)	.84	0.7 (0.4-1.1)^{a,b}	0.9 (0.5-1.3)^{,c}	1.0 (0.6-1.6)^{b,c}	.88^{a} .018^{b} .26^{c}
Legumes (serves/d)	0.1 (0.0-0.3)	0.0 (0.0-0.2)^{a,b}	0.1 (0.0-0.2)^{a,c}	0.2 (0.0-0.4)^{b,c}	.005^{a} <.001^{b} .077^{c}	0.1 (0.0-0.3)	0.1 (0.0-0.3)	0.1 (0.0-0.2)	.32	0.1 (0.0-0.3)	0.0 (0.0-0.2)	0.1 (0.0-0.3)	.40
Seafood (serves/d)	0.3 (0.2-0.5)	0.2 (0.1-0.4)^{a,b}	0.3 (0.2-0.5)^{a,c}	0.4 (0.2-0.6)^{b,c}	.003^{a} <.001^{b} .030^{c}	0.3 (0.2-0.5)	0.3 (0.1-0.4)	0.3 (0.2-0.5)	.73	0.3 (0.2-0.5)	0.3 (0.1-0.5)	0.3 (0.2-0.5)	.21
Poultry (serves/d)	0.3 (0.2-0.6)	0.3 (0.1-0.4)^{a,b}	0.3 (0.2-0.5)^{a,c}	0.4 (0.2-0.7)^{b,c}	.005^{a} <.001^{b} .049^{c}	0.4 (0.2-0.6)	0.3 (0.2-0.5)	0.3 (0.2-0.5)	.25	0.3 (0.2-0.5)	0.3 (0.2-0.6)	0.4 (0.2-0.6)	.65

Discretionary (serves/d)	17.2 (12.2-23.7)	19.4 (14.4-26.4)[a,b]	16.8 (12.4-23.3)[a,c]	14.2 (10.2-21.1)[b,c]	.026[a] <.001[b] .013[c]	11.5 (8.0-15.3)[a,b]	17.3 (13.6-21.0)[a,c]	25.3 (19.5-31.6)[b,c]	<.001[a,b,c]	16.0 (11.4-23.2)[a,b]	16.4 (12.4-22.5)[a,c]	18.5 (12.8-24.8)[b,c]	1.00[a] .030[b] .049[c]
Starchy vegetables (serves/d)	0.6 (0.3-1.0)	0.5 (0.3-0.8)[a,b]	0.6 (0.3-1.1)[a,c]	0.7 (0.3-1.3)[b,c]	<.001[a,b] .71[c]	0.3 (0.1-0.6)[a,b]	0.6 (0.3-0.9)[a,c]	1.0 (0.6-1.6)[b,c]	<.001[a,b,c]	0.5 (0.2-1.0)[a,b]	0.5 (0.3-1.0)[a,c]	0.7 (0.4-1.1)[b,c]	.49[a] <.001[b] .12[c]
Processed meats (serves/d)	0.1 (0.0-0.2)	0.1 (0.0-0.3)[a,b]	0.1 (0.0-0.3)[a,c]	0.1 (0.0-0.2)[b,c]	1.00[a] <.001[b] .001[c]	0.0 (0.0-0.1)[a,b]	0.1 (0.0-0.3)[a,c]	0.2 (0.1-0.4)[b,c]	<.001[a,b] .001[c]	0.1 (0.0-0.2)	0.1 (0.0-0.2)	0.1 (0.0-0.3)	.21
Fruit juice (serves/d)	0.0 (0.0-0.0)	0.0 (0.0-0.0)	0.0 (0.0-0.0)	0.0 (0.0-0.0)	.20	0.0 (0.0-0.0)[a,b]	0.0 (0.0-0.0)[a,c]	0.0 (0.0-0.0)[b,c]	.26[a] <.001[b] .086[c]	0.0 (0.0-0.0)	0.0 (0.0-0.0)	0.0 (0.0-0.0)	.69
Eggs (serves/d)	0.2 (0.1-0.3)	0.1 (0.1-0.2)[a,b]	0.1 (0.1-0.3)[a,c]	0.2 (0.1-0.3)[b,c]	.49[a] .003[b] .18[c]	0.1 (0.0-0.2)[a,b]	0.2 (0.1-0.3)[a,c]	0.2 (0.1-0.4)[b,c]	.004[a] <.001[b] .018[c]	0.1 (0.1-0.3)	0.2 (0.1-0.3)	0.2 (0.1-0.3)	.29
Red meats (serves/d)	1.1 (0.7-1.6)	1.0 (0.7-1.5)	1.2 (0.8-1.7)	1.2 (0.7-1.8)	.32	0.9 (0.5-1.3)[a,b]	1.1 (0.7-1.6)[a,c]	1.4 (1.0-2.0)[b,c]	.016[a] <.001[b]	1.3 (0.8-1.9)[a,b]	1.0 (0.7-1.5)[a,c]	1.0 (0.7-1.6)[b,c]	.005[a] .004[b] 1.00[c]

									.003[c]				
Cheese (serves/d)	0.3 (0.1-0.6)	0.3 (0.2-0.6)[a,b]	0.4 (0.1-0.7)[a,c]	0.3 (0.1-0.6)[b,c]	.35[a] 1.00[b] .038[c]	0.2 (0.0-0.4)[a,b]	0.3 (0.2-0.6)[a,c]	0.5 (0.2-0.8)[b,c]	<.001[a,b] .049[c]	0.4 (0.1-0.7)	0.4 (0.1-0.7)	0.2 (0.1-0.5)	.065
Milk alternatives (serves/d)	0.0 (0.0-0.0)	0.0 (0.0-0.0)[a,b]	0.0 (0.0-0.0)[a,c]	0.0 (0.0-0.0)[b,c]	1.00[a] <.001[b] .003[c]	0.0 (0.0-0.0)[a,b]	0.0 (0.0-0.0)[a,c]	0.0 (0.0-0.0)[b,c]	.005[a] .001[b] 1.00[c]	0.0 (0.0-0.0)	0.0 (0.0-0.0)	0.0 (0.0-0.0)	.23
Organ meats (serves/d)	0.0 (0.0-0.0)	0.0 (0.0-0.0)	0.0 (0.0-0.0)	0.0 (0.0-0.0)	.50	0.0 (0.0-0.0)	0.0 (0.0-0.0)	0.0 (0.0-0.0)	.76[a] .25[b] .020[c]	0.0 (0.0-0.0)[a,b]	0.0 (0.0-0.0)[a,c]	0.0 (0.0-0.0)[b,c]	.020[a] .76[b] .26[c]
Wholegrains (serves/d)	1.9 (0.9-3.1)	1.8 (1.0-2.9)	2.0 (0.9-3.0)	1.9 (0.8-3.2)	.66	1.7 (0.9-3.0)	1.8 (0.9-2.9)	2.2 (0.9-3.3)	.30	0.7 (0.1-1.4)[a,b]	1.9 (1.3-2.7)[a,c]	3.4 (2.6-4.3)[b,c]	<.001[a,b,c]
Refined grains (serves/d)	2.7 (1.6-4.1)	2.7 (1.8-4.0)	2.5 (1.6-3.9)	2.9 (1.6-4.6)	.20	2.2 (1.3-3.9)[a,b]	2.7 (1.8-4.0)[a,c]	3.1 (2.0-4.5)[b,c]	.069[a] .002[b] .077[c]	4.2 (3.0-6.1)[a,b]	2.5 (1.7-3.5)[a,c]	1.7 (1.0-2.6)[b,c]	<.001[a,b,c]
Milk (serves/d)	1.1 (0.6-1.7)	1.3 (0.7-2.0)[a,b]	1.0 (0.6-1.6)[a,c]	0.9 (0.4-1.5)[b,c]	<.001[a,b] .45[c]	0.9 (0.4-1.4)[a,b]	1.0 (0.6-1.7)[a,c]	1.3 (0.8-2.0)[b,c]	.16[a] <.001[b] .006[c]	0.7 (0.3-1.2)[a,b]	1.0 (0.6-1.6)[a,c]	1.5 (1.1-2.3)[b,c]	.003[a] <.001[b,c]

Yoghurt (serves/d)	0.0 (0.0-0.1)	0.0 (0.0-0.0)[a,b]	0.0 (0.0-0.1)[a,c]	0.0 (0.0-0.3)[b,c]	.24[a] .001[b] .19[c]	0.0 (0.0-0.2)	0.0 (0.0-0.2)	0.0 (0.0-0.0)	.27	0.0 (0.0-0.0)[a,b]	0.0 (0.0-0.2)[a,c]	0.0 (0.0-0.4)[b,c]	<.001[a,b] .003[c]
Soy products (serves/d)	0.0 (0.0-0.0)	0.0 (0.0-0.1)[a,b]	0.0 (0.0-0.0)[a,c]	0.0 (0.0-0.1)[b,c]	.24[a] .002[b] .22[c]	0.0 (0.0-0.0)[a,b]	0.0 (0.0-0.0)[a,c]	0.0 (0.0-0.0)[b,c]	1.00[a] <.001[b] .006[c]	0.0 (0.0-0.0)	0.0 (0.0-0.0)	0.0 (0.0-0.0)	.12
Other fruits (serves/d)	1.4 (0.8-2.1)	1.1 (0.6-1.7)[a,b]	1.5 (0.9-2.2)[a,c]	1.5 (1.0-2.4)[b,c]	<.001[a,b] 1.00[c]	1.4 (0.7-2.2)	1.3 (0.7-2.0)	1.4 (0.8-2.2)	.90	0.9 (0.6-1.7)[a,b]	1.4 (0.9-2.1)[a,c]	1.7 (1.0-2.6)[b,c]	<.001[a,b] .12[c]
Citrus, melons and berries (serves/d)	0.3 (0.1-0.6)	0.2 (0.0-0.6)	0.3 (0.1-0.6)	0.3 (0.1-0.6)	.061	0.3 (0.1-0.7)	0.3 (0.1-0.6)	0.2 (0.0-0.5)	.051	0.2 (0.0-0.4)[a,b]	0.3 (0.1-0.7)[a,c]	0.4 (0.1-0.8)[b,c]	.001[a] <.001[b] .077[c]
Nuts and seeds (serves/d)	0.2 (0.0-0.6)	0.1 (0.0-0.5)[a,b]	0.1 (0.0-0.7)[a,c]	0.3 (0.0-0.8)[b,c]	1.00[a] .001[b] .030[c]	0.2 (0.0-0.7)	0.2 (0.0-0.6)	0.2 (0.0-0.6)	.79	0.0 (0.0-0.4)[a,b]	0.2 (0.0-0.7)[a,c]	0.3 (0.0-0.8)[b,c]	<.001[a,b] .030[c]

[1]*P* values were obtained using the median test and Bonferroni correction for multiple tests to compare all dietary pattern score tertile groups for differences in median values of continuous variables. Differences between groups are denoted by each letter a, b, or c. [†]No differences between groups were observed after Bonferroni correction for multiple tests.

Supplementary Table 3. Median (interquartile range) daily dietary pattern scores and according to transitions in frailty status from robust at baseline (n = 296)

Dietary pattern factor	Frailty status at 3-year follow-up		
	Robust (n=125)	Pre-frail (n=155)	Frail (n=16)
Factor 1: 'Vegetables-legumes-seafood'	0.06 (-0.45, 0.69)	-0.10 (-0.63, 0.44)	-0.26 (-0.64, 0.22)
Factor 2: 'Discretionary-starchy vegetables-processed meats'	-0.03 (-0.71, 0.48)	-0.25 (-0.77, 0.54)	-0.17 (-0.72, 0.37)
Factor 3: 'Wholegrains-milk-other fruits'	0.13 (-0.58, 0.86)	-0.02 (-0.82, 0.50)	-0.02 (-0.88, 0.71)

Supplementary Table 4. Median (interquartile range) daily dietary pattern scores and according to transitions in frailty status from pre-frail at baseline (n = 273)

Dietary pattern factor	Frailty status at 3-year follow-up		
	Robust (n=22)	Pre-frail (n=178)	Frail (n=73)
Factor 1: 'Vegetables-legumes-seafood'	0.23 (-0.33, 0.66)	-0.18 (-0.56, 0.36)	-0.28 (-0.70, 0.35)
Factor 2: 'Discretionary-starchy vegetables-processed meats'	-0.21 (-1.01, 0.50)	-0.12 (-0.63, 0.48)	0.07 (-0.47, 0.73)
Factor 3: 'Wholegrains-milk-other fruits'	0.15 (-0.55, 0.81)	0.01 (-0.62, 0.53)	-0.03 (-0.55, 0.75)

Supplementary Table 5. Longitudinal associations between dietary pattern factor scores, incident robust and incident frailty from pre-frail using multinomial logistic regression presented as odds ratios (n = 273)

**Dietary Pattern Factor Scores**	Bottom tertile (reference category)	Middle tertile	Top tertile	As continuous variable (per 1 increment)
Factor 1: ‘Vegetables-legumes-seafood’[a]				
**Robust**				
Model 1	1	1.33 (0.36, 4.96) P = .67	2.57 (0.78, 8.44) P = .12	1.15 (0.76, 1.75) P = .51
Model 2	1	1.26 (0.33, 4.85) P = .74	2.56 (0.76, 8.66) P = .13	1.07 (0.70, 1.63) P = .77
Model 3	1	1.24 (0.32, 4.86) P = .76	2.06 (0.58, 7.36) P = .27	1.03 (0.63, 1.66) P = .91
**Frailty**				
Model 1	1	0.92 (0.48, 1.78) P = .81	0.75 (0.38, 1.49) P = .42	0.86 (0.63, 1.17) P = .33
Model 2	1	0.84 (0.42, 1.68) P = .62	0.71 (0.35, 1.46) P = .35	0.79 (0.56, 1.11) P = .18
Model 3	1	0.95 (0.45, 1.90) P = .88	0.89 (0.41, 1.91) P = .76	0.85 (0.60, 1.20) P = .35
Factor 2: ‘Discretionary-starchy vegetables-processed meats’[b]				
**Robust**				
Model 1	1	0.56 (0.19, 1.64) P = .29	0.54 (0.19, 1.59) P = .26	0.78 (0.48, 1.27) P = .31
Model 2	1	0.52 (0.17, 1.61) P = .26	0.44 (0.12, 1.64) P = .22	0.72 (0.41, 1.26) P = .72
Model 3	1	0.51 (0.15, 1.71) P = .27	0.33 (0.07, 1.44) P = .14	0.62 (0.34, 1.14) P = .12
**Frailty**				
Model 1	1	1.30 (0.64, 2.63) P = .47	1.51 (0.76, 2.99) P = .24	1.20 (0.90, 1.62) P = .22
Model 2	1	1.16 (0.56, 2.43)	1.25 (0.54, 2.88)	1.11 (0.77, 1.60)

		P = .69	P = .61	P = .58
Model 3	1	1.34 (0.60, 2.96)	1.41 (0.57, 3.49)	1.14 (0.76, 1.70)
		P = .48	P = .46	P = .53
Factor 3: ‘Wholegrains-milk-other fruits’[c]				
**Robust**				
Model 1	1	0.67 (0.20, 2.22)	1.59 (0.57, 4.46)	1.34 (0.85, 2.10)
		P = .51	P = .38	P = .21
Model 2	1	0.63 (0.18, 2.20)	1.57 (0.53, 4.65)	1.38 (0.85, 2.25)
		P = .47	P = .42	P = .20
Model 3	1	0.65 (0.16, 2.68)	2.15 (0.65, 7.12)	1.54 (0.93, 2.57)
		P = .55	P = .21	P = .096
**Frailty**				
Model 1	1	1.02 (0.52, 1.99)	1.21 (0.62, 2.37)	1.10 (0.83, 1.46)
		P = .96	P = .58	P = .50
Model 2	1	0.79 (0.39, 1.62)	1.03 (0.50, 2.12)	1.07 (0.79, 1.45)
		P = .53	P = .94	P = .66
Model 3	1	0.81 (0.38, 1.72)	1.03 (0.48, 2.23)	1.11 (0.80, 1.54)
		P = .58	P = .94	P = .54

Notes: Model 1 unadjusted (n = 273 for total, 22 robust, 178 pre-frail, and 73 frail); Model 2 adjusted by sociodemographic and lifestyle factors (age (continuous), BMI (continuous), smoking status (nonsmoker v. ex-smoker v. current smoker), energy intake (continuous), and supplement use including vitamins, minerals, and/or fish oil (yes v. no)) (n = 271 for total, 22 robust, 177 pre-frail, and 72 frail); Model 3 adjusted by Model 2 plus health (haemoglobin (continuous), number of medications (continuous), number of comorbidities (continuous), and self-rated health (very poor/poor/fair v. good/excellent)) (n = 261 for total, 20 robust, 173 pre-frail, and 68 frail)

[a] Bottom tertile ≤-0.47, n = 83; middle tertile -0.46-0.17, n = 93; top tertile ≥0.18, n = 97

[b] Bottom tertile ≤-0.48, n = 98; middle tertile -0.47-0.31, n = 91; top tertile ≥0.32, n = 84

[c] Bottom tertile ≤-0.41, n = 89; middle tertile -0.40-0.41, n = 94; top tertile ≥0.42, n = 90
